# Supplementary material for: Predicting wavelength-dependent photochemical reactivity and selectivity
Source: Nat Commun. 2021 Mar 16;12:1691. doi: 10.1038/s41467-021-21797-x (PMC7966369; doi:10.1038/s41467-021-21797-x)
Supplement: Supplementary file 1 — Supplementary Information [file 41467_2021_21797_MOESM1_ESM.pdf]

Supplementary Information

Predicting Wavelength-Dependent Photochemical Reactivity and Selectivity

Jan P. Menzel,[1,2,3] Benjamin B. Noble,[4] James P. Blinco[*,1,2] and Christopher Barner-Kowollik[*,1,2]

[1]School of Chemistry and Physics
Queensland University of Technology (QUT)
Brisbane, QLD 4000, Australia

[2]Centre for Materials Science
Queensland University of Technology (QUT)
Brisbane, QLD 4000, Australia

[3]Centre for Data Science
Queensland University of Technology (QUT)
Brisbane, QLD 4000, Australia

[4]School of Engineering, College of Science, Engineering and Health
RMIT University
Melbourne, VIC 3001, Australia

Table of Contents

1. Experimental Procedures 3
1.1 Materials 3
1.2 High resolution electrospray ionization mass spectrometry 3
1.3 Nuclear magnetic resonance spectroscopy 3
1.4 Ultraviolet visible spectroscopy 3
1.4 Photoreactions with light emitting diodes (LEDs) 4
1.5 Tunable laser photoreactions 8
1.6 Transmittance of glass vials 8
1.7 UV Vis spectra 11
2 Results and Discussion 14
2.1 Quantitative evaluation of electrospray ionization mass spectra of A and AP 14
2.2 Quantum yields of photoenol ligation 19
2.2.1 Wavelength dependence 19
2.2.2 Concentration dependence 22
2.3 Curve fitting for wavelength and concentration dependent quantum yields 25
2.4 Evidence for intramolecular noncovalent interactions between oxygen and sulfur in A causing conformers and their influence on wavelength-dependent quantum yields 28
2.4.1 Comparison of UV Vis spectra of relevant derivatives of A and solvent dependence of absorbance 28
2.4.2 Wavefunction and density functional theory calculations 29
2.5 Simulation of LED light induced photoreactions and prediction of conversion 32
2.5 Simulation of wavelength-dependent selectivity of competing photoreactions 34
2.6 Conversion of photoligation of A with NEM using LED 2 and LED 3 35
2.7 Conversion of photoligation of A with NEM in the presence of HNBA using LED 2 37
2.8 Additional predictions and experiments with LED 1 and LED 4 39
2.9.1 Sequence-independent λ-orthogonal photoligation employing two photoenol ligation reactions 42
2.9.2. Quantum yields of 2-(dodecylthio)-6-methylbenzaldehyde C and unprecedented selectivity between C and tetrazole D 45
2.9.3. Prediction and experimental observation of selectivity between tetrazole D and *o*-methylbenzaldehyde B 48
3 Computational methodology (density functional theory) 51
Other Supplementary Data and Files 55
Author Contributions 55
References 55

1. Experimental Procedures

1.1 Materials

Acetonitrile (extra dry, over molecular sieve, 99.9%, Acros Organics), acetonitrile-d_3 (99.8 atom% D, Cambridge Isotope Laboratories, Inc.), methanol (>99.9%, (LC/MS), Sigma Aldrich), *N*-ethylmaleimide (>98% (HPLC), Sigma Aldrich), 2-hydroxy-5-nitrobenzaldehyde (98%, Sigma Aldrich), tetrahydrofuran (99.5%, Sigma Aldrich), sodium trifluoroacetate (>99% (HPLC), Fluka) were used as received.
The preparation of **A** and **B** was reported previously.[1,2]

1.2 High resolution electrospray ionization mass spectrometry

ESI mass spectra were recorded on a Q-Exactive (Orbitrap) mass spectrometer (Thermo Fisher Scientific, San Jose, CA, USA) equipped with an HESI II probe. The instrument was calibrated in the *m/z* range 74-1822 using premixed calibration solutions (Thermo Scientific). A constant spray voltage of 4.7 kV and a dimensionless sheath gas of 5 were applied. The capillary temperature and the S-lens RF level were set to 320 °C and 62.0, respectively. The samples were dissolved with a concentration of 0.05 mg mL^{-1} in a mixture of THF and MeOH (3:2) containing 100 mmol L^{-1} of sodium trifluoroacetate and infused with a flow of 5 µL min^{-1}. Data were processed with Xcalibur, mMass – Open Source Mass Spectrometry Tool, Microsoft Excel and OriginPro 9.1G.

1.3 Nuclear magnetic resonance spectroscopy

^{1}H-NMR spectra were recorded with a Bruker 600 at 600 MHz. Samples were dissolved in CD_3CN. Spectra of irradiated solutions or of the stock solution were recorded with a D1 time of 2 seconds and a scan number of 128 scans. The *δ*-scale was referenced to the signal of residual solvent (CHD_2CN). Data were processed with MestReNova and OriginPro 9.1G.

1.4 Ultraviolet visible spectroscopy

UV Vis spectra were recorded with a Shimadzu UV-2700 spectrophotometer equipped with a CPS-100 electronic temperature control cell positioner. The temperature is set to 20˚C. For each sample, a baseline was acquired using the same cuvette (*Hellma Analytics* quartz high precision cell with a path length of 10 mm at ambient temperature) and solvent (3 mL solvent). Samples were dissolved in 3 mL solvent and spectra were acquired from 600 nm to 250 nm.

1.4 Photoreactions with light emitting diodes (LEDs)

Samples were generally prepared in or transferred to 0.8 mL clear glass vials (Supelco, 7 x 40 mm, flat bottom, 24738-U), crimped (8 mm crimp seal with Teflon® faced red rubber, Supelco, Lot: 109143), and deoxygenated by a stream of nitrogen gas for 10 minutes prior to irradiation. The sample solution inside the glass vials is irradiated with LED light from below, while the sides of the glass vial are covered with aluminum foil to minimize the loss of light. The solutions are stirred with a magnetic stir bar at 500 rpm. The irradiation setup for the LEDs is shown in Figure S1. The glass vial is placed in a custom-made 3D printed holder (see Figure S1). The distance from the LED to the bottom of the glass vial is 2 mm. The LED is mounted on a heat sink, which is sitting on a fan as shown in Figure S1. The measured output power of the LEDs as used here is shown in Table S1 and Table S2, if not described differently. The measurement was performed with a Coherent FieldMate Laser Power Meter, Model PM2. This power meter has a circular sensor area with a diameter of 19 mm. As the irradiated area of the bottom of the sample glass vial is circular with a diameter of 8 mm, a 3D printed LED detector scaffold containing a hole with a diameter of 8 mm used, refer also to Figure S2. The dimensions between the LED and both the 3D printed LED precision batch photoreactor as well as the LED detector scaffold are equal. This ensures that the power measured by the detector corresponds to the irradiation conditions in the experiment. Emission spectra of the LEDs are shown in Figure S3. LED emission spectra were measured with an Ocean Optics Miniature Spectrometer FLAME-T-UV-VIS.

Table S1: Overview of the LEDs used in this work. The output power is dependent on the voltage and current that the LED is operated with: For the measurement of the output power, the respective settings were applied on the regulated power supply. Column six contains the average of the measured output power for each wavelength and the associated standard deviation, refer also to Table S2.

LED	λ_{max}	Description of LED; manufacturer	Further details of LED	Voltage and current (regulated power supply)	Measured output power of LED
1	285 nm	UVTOP285 DEEP UV LED; QPhotonics	Hemispherical lens for collimated emission (6 deg.)	U = 6.8 V; I = 0.03 A	1.0 ± 0.2 mW
2	343 nm	LED SMD6363, CUD4AF1B	Emission angle approx. 110 deg.	U = 3.7 V; I = 0.075 A	5.3 ± 0.1 mW
3	380 nm	EPILED ultraviolet; Bridgelux	Emission angle approx. 120 deg.	U = 3.2 V; I = 0.075 A	37.9 ± 0.6 mW
5	440 nm	Royal Blue 3 W High Power LED	Emission angle approx. 120 deg.	U = 2.68 V; I = 0.020 A	14.1 ± 0.8 mW

Table S2: Measured output power of LEDs, see also Table S1.

Measurement number	LED 1 P / mW	LED 2 P / mW	LED 3 P / mW	LED 4 P / mW
1	1.0	5.3	38.4	14.1
2	0.9	5.4	37.4	14.4
3	1.2	5.5	37.0	13.0
4	0.8	5.2	38.2	12.9
5	1.3	5.3	38.4	14.9
6	1.2	5.0	37.8	13.9
7	0.8	5.3	37.3	14.1
8	0.7	5.1	38.3	14.0
9	0.9	5.2	38.8	15.2
10	1.1	5.2	37.7	14.9

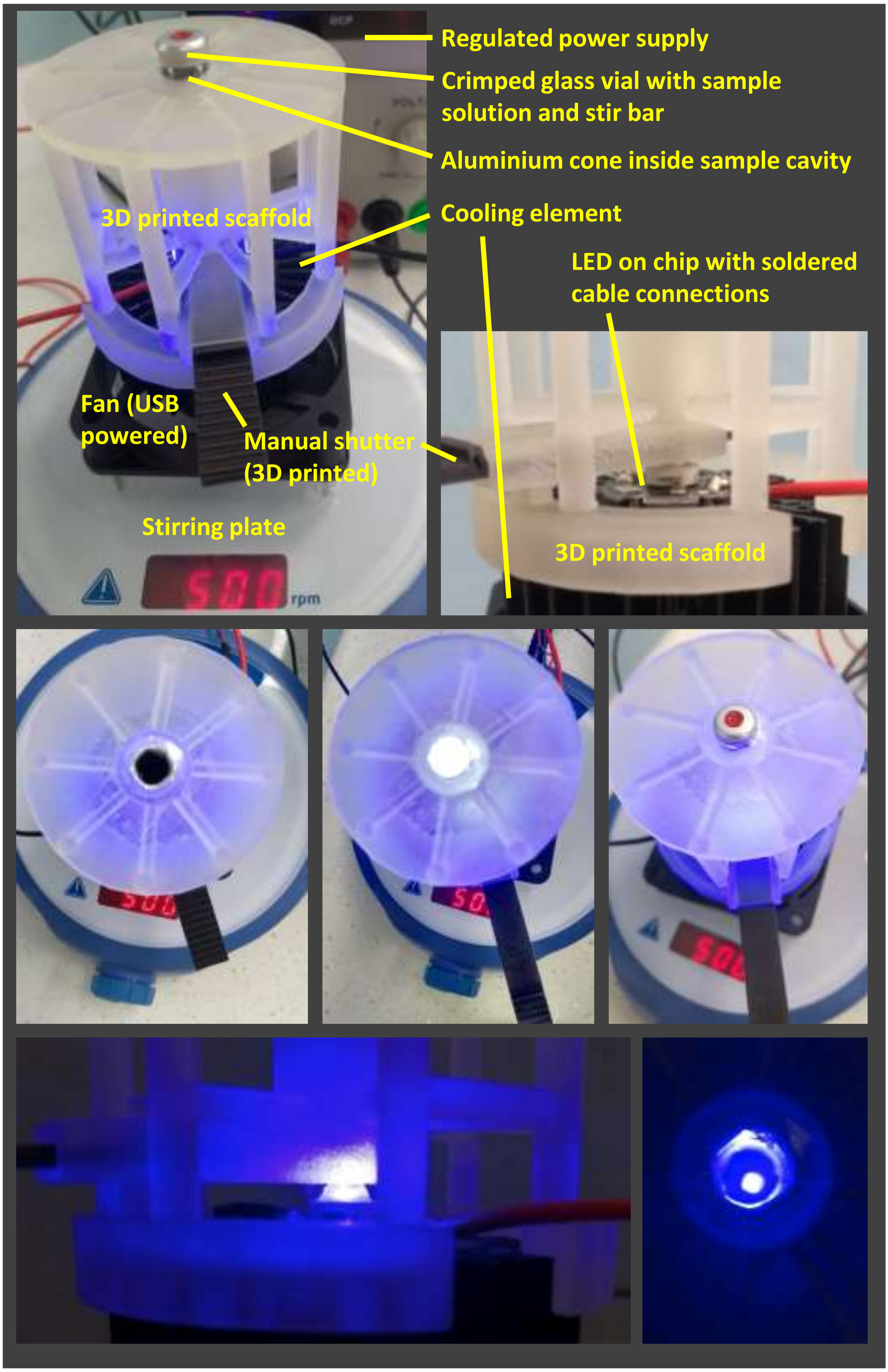

Figure S1: Irradiation setup with the 3D printed batch photoreactor. The reactor is placed on a cooling element on which the light emitting diode is mounted. A fan is installed for further cooling. The setup is placed on a stirring plate. An aluminium cone is fitted inside the sample cavity for an increased efficiency of the reactor.

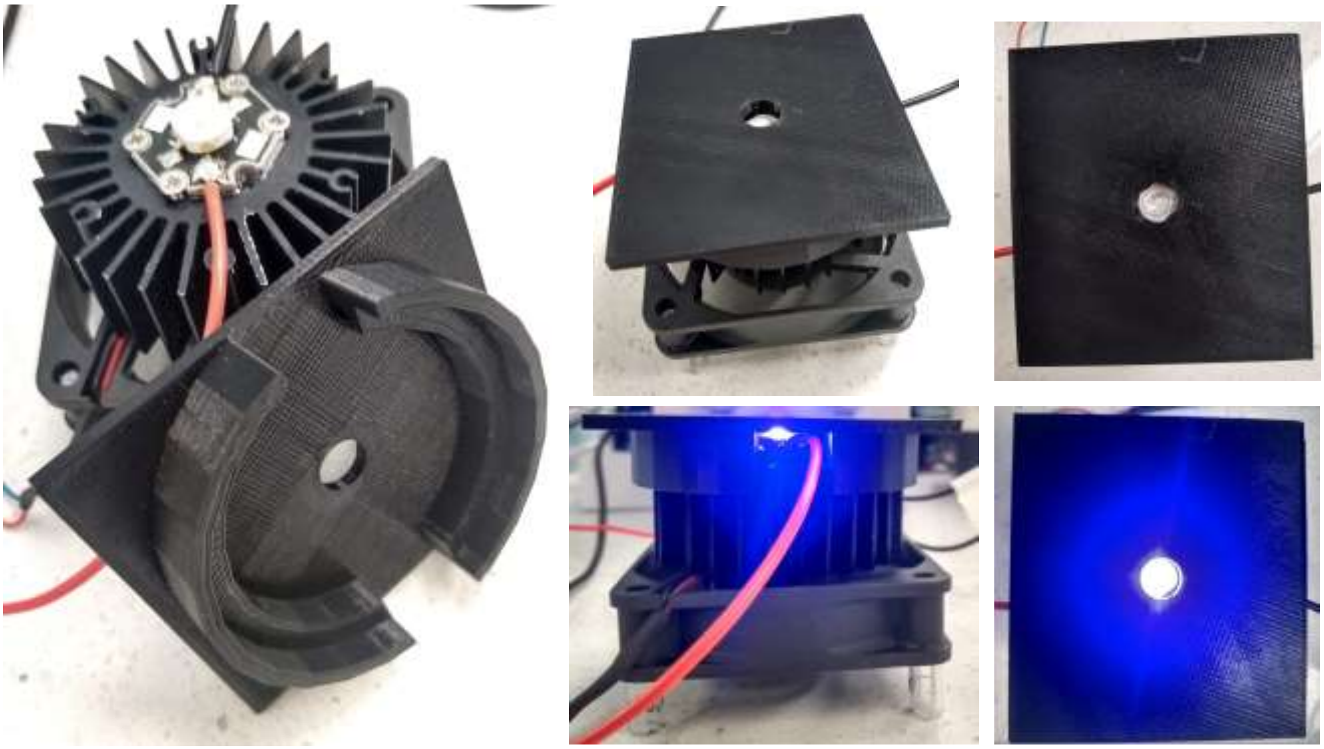

Figure S2: Detector scaffold for measurement of LED power. Left: 3D printed detector scaffold and LED 4, mounted on a fan. Right: Setup for measuring the output with the detector scaffold. The power meter is held in place on top of the detector scaffold, so that light passing through the hole reaches the power meter.

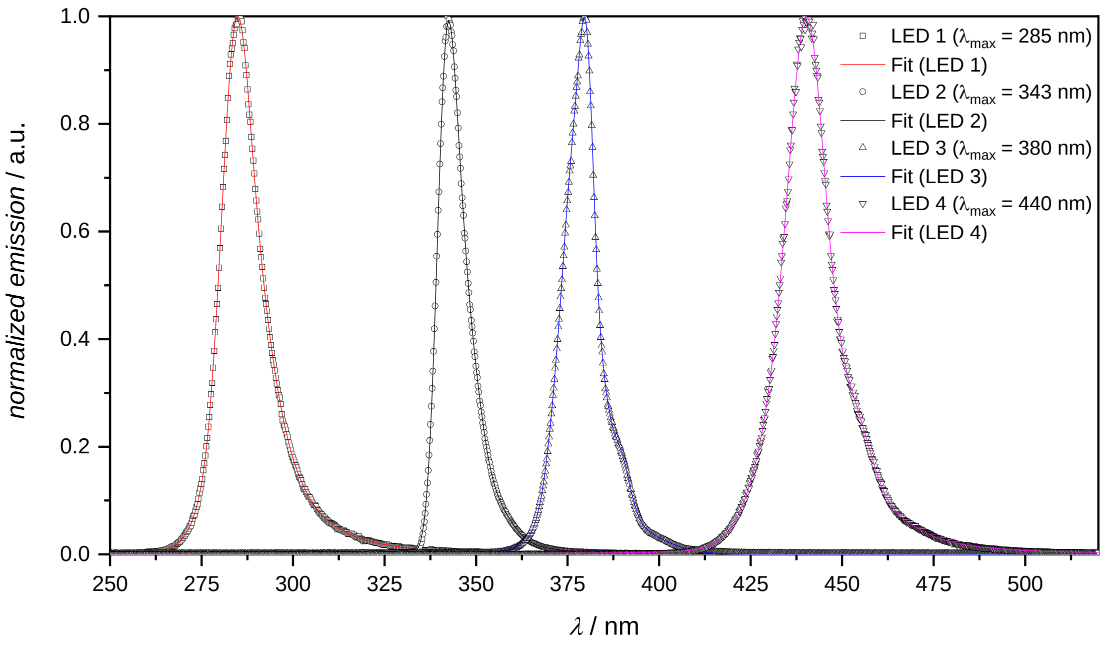

Figure S3: LED emission spectra. Normalized emission spectra and respective fitted function of the light emitting diodes employed in this work.

The emission spectra were each fitted with equation [1], yielding an analytical expression that is used in the numerical simulation. The fit parameters for equation [1] are shown in Table S2-5 for each spectrum, respectively. Figure S3 shows both each spectrum as obtained as well as the respective fit.

$$y = ae^{-\left(\frac{(x-b)^2}{2c^2}\right)} + de^{-\left(\frac{(x-s)^2}{2f^2}\right)} + ge^{-\left(\frac{(x-h)^2}{2i^2}\right)} + je^{-\left(\frac{(x-k)^2}{2l^2}\right)} + me^{-\left(\frac{(x-n)^2}{2o^2}\right)} + pe^{-\left(\frac{(x-q)^2}{2r^2}\right)} \quad [1]$$

Table S3: Fit parameters for equation [1], representing the emission spectrum of each LED, respectively. The fitted curve is shown in Figure S3. In the case of LED 1, only the data in the wavelength range of 250 nm – 350 nm was used to calculate the fitted function.

parameter	LED 1 (λ_{max} = 285 nm)	LED 2 (λ_{max} = 343 nm)	LED 3 (λ_{max} = 380 nm)	LED 4 (λ_{max} = 440 nm)
a	0.79979	0.80766	0.61777	0.60582
b	284.15613	341.6736	380.35059	440.58471
c	3.86295	2.57945	2.07071	4.68460
d	0.23203	0.2413	0.08317	0.37022
s	290.19406	345.0521	383.55166	437.92114
f	4.23537	2.44903	11.74498	8.50065
g	0.09949	0.27731	0.13335	0.00356
h	294.71877	348.0167	384.76841	563.53413
i	5.79419	3.37941	1.87352	198.82567
j	0.02333	0.01315	0.11725	0.13518
k	305.96032	362.7097	389.01515	453.17793
l	21.05534	9.03913	2.70496	4.84526
m	0.08454	0.09035	0.17849	0.04145
n	293.96335	353.5456	376.64049	459.95325
o	11.86232	5.28352	2.19398	8.59983
p	0.05864	0.0012	0.44245	0.03431
q	276.94418	364.7458	375.51123	453.97177
r	4.04295	14.46467	4.06981	21.76607

1.5 Tunable laser photoreactions

"Tunable laser experiments with a defined quantity of incident photons were carried out with an Opotek Tunable Laser System Opolette. An optical parametric oscillator (OPO) was pumped with a diode pumped Nd:YAG laser (repetition rate 20 Hz). The beam is redirected into the vertical cylindrical hole of a custom-made sample holder, refer to Figure S4. The energy of the incident laser pulses was measured by an Energy Max PC power meter (Coherent) directly above the sample holder. Prism and sample holder are positioned in a way that the complete diameter of the hole of the sample holder is covered by the incident laser beam." (Adapted and modified from our previous publications[3,4]). Samples are prepared analogously to the LED experiments (also using the same glass vials).

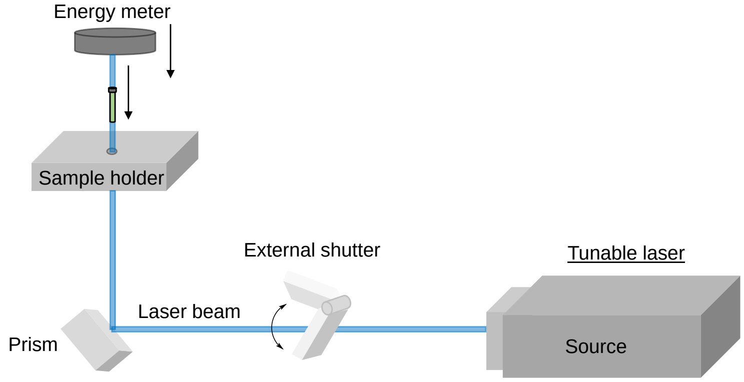

Figure S4: Schematic representation of the tunable laser setup. The beam exiting the laser is redirected by a prism into the sample holder. The irradiation time is controlled by an external shutter. (Adapted and modified from our previous publications[3,4]).

Control over the incident number of photons in a tunable laser experiment

The number of photons $n_{p,\ pulse}$ ($[n_p]$ = mol) that a monochromatic laser pulse contains can be calculated by application of the Planck-Einstein relation from the energy of the pulse E_{pulse}, the incident wavelength λ, Planck's constant h and the speed of light c.

$$n_p = \frac{E_{pulse}\lambda}{h\ c\ N_A} \qquad [2]$$

Irradiation procedure with known photon count

Prior to each irradiation sequence the respective (stock) solution was deoxygenated by a stream of nitrogen for 10 minutes. The tunable laser, including the pump source, was started up to 30 minutes before irradiation to allow the energy output of the laser to stabilize. The direction of the beam was controlled by adjusting the orientation of the prism. The whole cross-sectional area of the sample is irradiated by the laser beam. The intensity of the beam was monitored and adjusted. This general procedure is used for all tunable laser experiments performed for the determination of quantum yields. Using equation [2], the number of photons reaching the glass vial containing the sample can be calculated. The number of photons that reach the sample solution is calculated with the transmittance of the bottom part of the glass vials:

$$n_{p,sample} = T_\lambda * n_{p,pulse} \qquad [3]$$

1.6 Transmittance of glass vials

The transmittance of glass vials at varied wavelength was determined according to a previously published method.[2] The same glass vials and cut bottoms of glass vials as used previously are also used here.[1] Figure S5 shows the measured transmittance values for wavelengths in the range of 272 nm to 550 nm and a fitted curve. This analytical function [4] (with fit parameters as shown in Table S6) was fitted to the measured data in order to facilitate the use of the wavelength dependent transmittance data in the wavelength resolved numerical simulation. The transmittance at wavelengths shorter than 275 nm could not be measured, as the transmitted light at 270 nm was below the detection limit of the power meter. Thus, transmittance below 271 nm is assumed to be negligible. In order to estimate the confidence of the computational prediction that is done using the data described below, the error of the measurement of transmittance is taken into

account, refer to section 2.5 of this Supplementary Information. The average of the standard deviation of the transmittance values in the range of 320 nm to 430 nm is determined as 3.2.

Table S4: Transmittance of the bottom of the glass vials used in this study. The transmittance values shown and used here were obtained analogously to a method reported previously.[2] The glass vials were cut at a height of 3 mm. Thus, the number of photons delivered into the sample solution can be determined more precisely, than in initial attempts to estimate the number of photons.[3] The values are in agreement to the previously found transmittance at 285 nm, which was determined with the same glass vials as used here.[1] The transmittance values shown here are the average of three individual measurements for each wavelength. The standard deviation is calculated from the three values, which are not shown here.

λ / nm	T_{λ} / %	Standard deviation
275	10.23419	0.74982
280	18.25024	0.94703
285	28.31044	2.14137
290	37.43074	2.49132
295	49.11881	2.55629
300	58.57746	0.79725
310	68.34923	2.82282
320	74.95204	3.14492
330	81.62937	4.49571
340	79.27099	2.93382
350	82.52063	4.45387
360	83.15709	3.42489
370	83.40189	4.93431
380	83.17113	1.63659
390	85.48502	1.99615
400	85.31216	2.03069
410	86.83816	4.06669
430	85.71046	2.57226
450	85.49291	4.38069
475	83.84506	2.73637
500	82.44994	2.85985
550	83.10058	2.33119

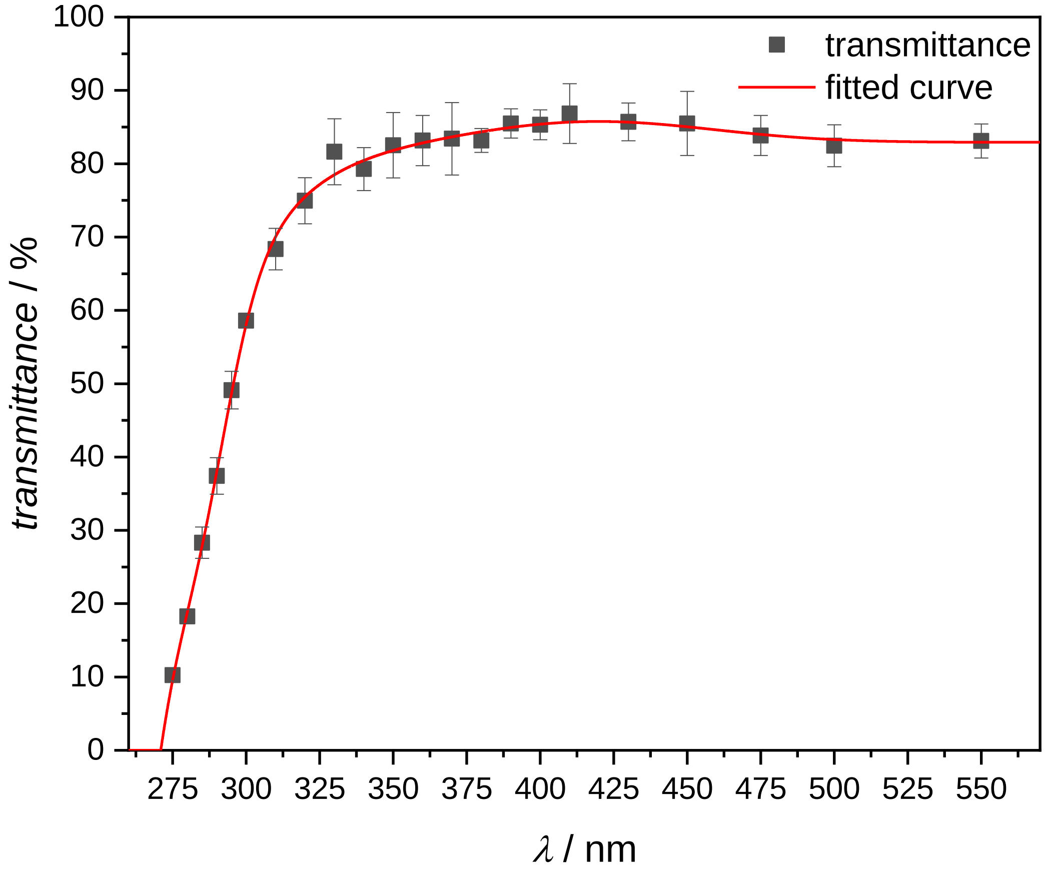

Figure S5: Transmittance of glass vials. Measured transmittance of the bottom of glass vials used in this work as shown in Table S4 and a fitted curve. The equation of the fitted curve is shown below and the parameters for the fit are shown on Table S5. The values shown are averages of three measurements and the error bar shows the standard deviation of these values.

$$y = \frac{\sqrt{(q-\lambda)^2} - (q-\lambda)}{2*(\lambda - q)} * \left(a - ce^{\frac{b-x}{d}} + ke^{-\left(\frac{(x-l)^2}{2m^2}\right)} + ne^{-\left(\frac{(x-o)^2}{2p^2}\right)}\right) \quad [4]$$

Table S5: Fit parameters for equation [4]. The fitted curve is shown in Figure S5.

parameter	value
a	82.91639
b	349.11562
c	1.91056
d	21.22769
k	-15.89814
l	284.58711
m	10.61099
n	2.91878
o	417.92409
p	40.68863
q	270.935

1.7 UV Vis spectra

UV Vis spectra shown here are recorded or selected from previously published work using small molecules to increase the accuracy of the molar attenuation coefficients.

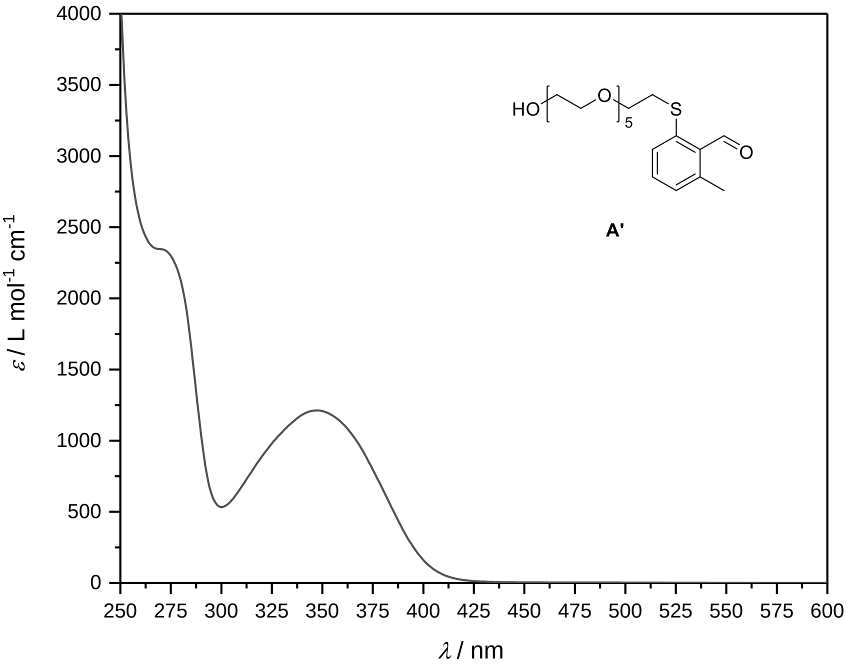

Figure S6: UV/Vis spectrum of A. UV / Vis spectrum of 2-((17-hydroxy-3,6,9,12,15-pentaoxaheptadecyl)thio)-6-methylbenzaldehyde A' measured in acetonitrile at 20°C.[5]

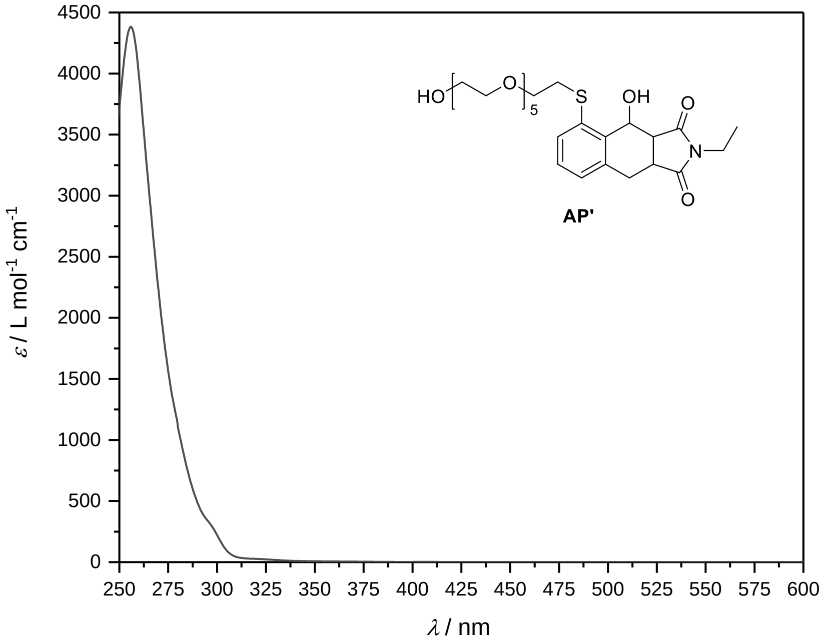

Figure S7: UV/Vis spectrum of AP'. UV / Vis spectrum of 2-ethyl-4-hydroxy-5-((17-hydroxy-3,6,9,12,15-pentaoxaheptadecyl)thio)-3a,4,9,9a-tetrahydro-1*H*-benzo[*f*]isoindole-1,3(2*H*)-dione AP', measured in acetonitrile at 20°C.[5]

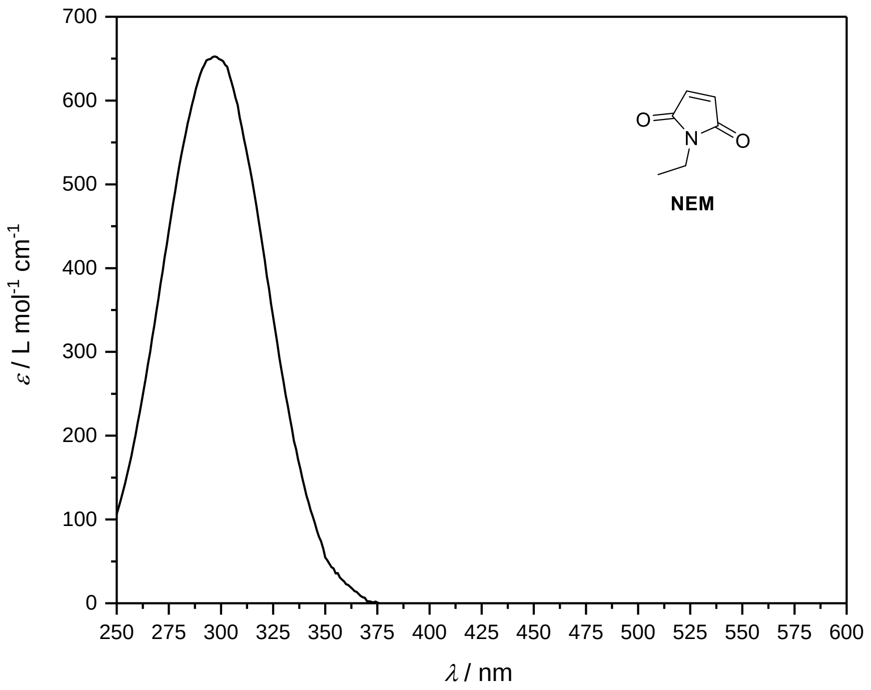

Figure S8: UV/Vis spectrum of NEM. UV / Vis spectrum of *N*-ethylmaleimide NEM in acetonitrile at 20°C.[1]

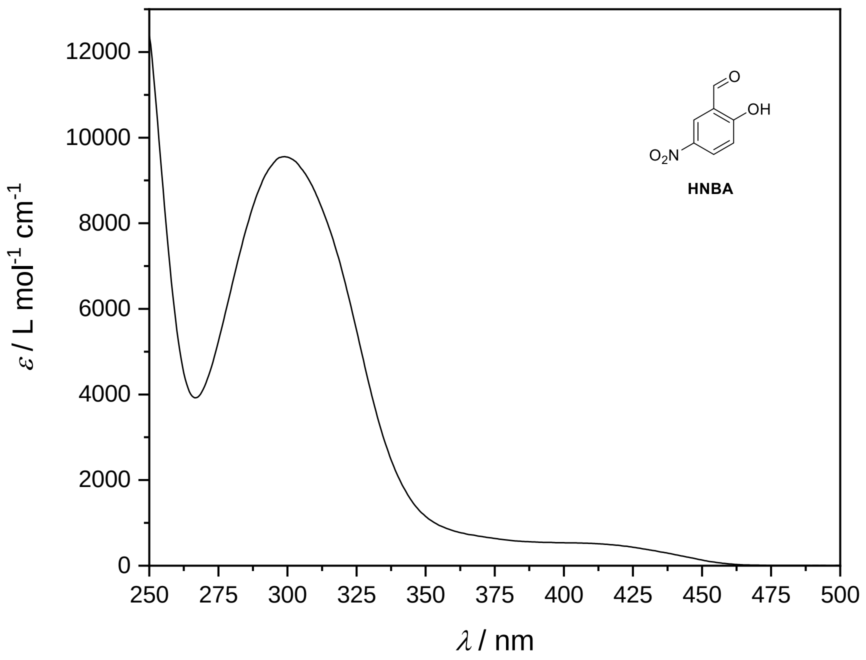

Figure S9: UV/Vis spectrum of HNBA. UV / Vis spectrum of 2-hydroxy-5-nitrobenzaldehyde HNBA in acetonitrile at 20°C.

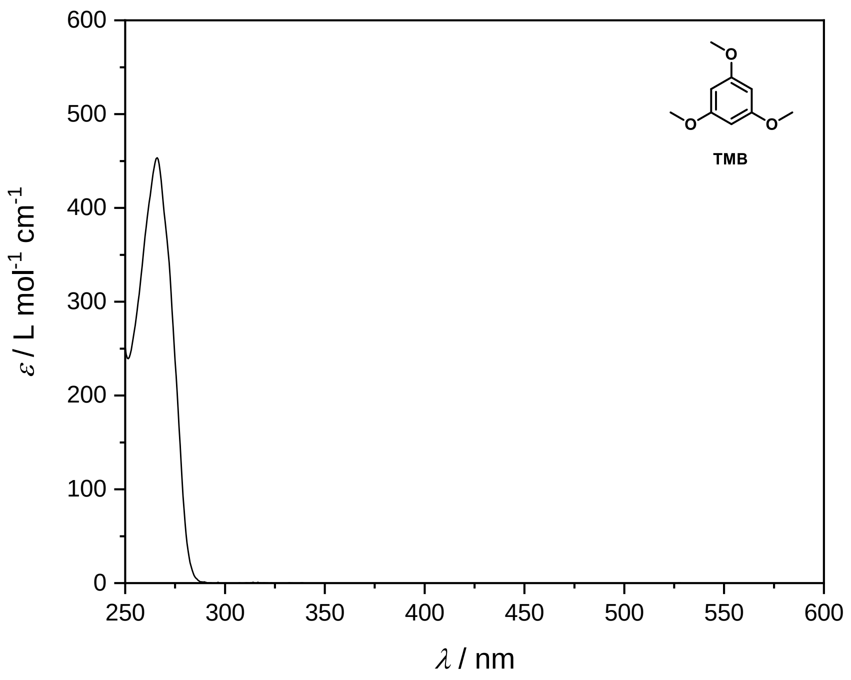

Figure S10: UV/Vis spectrum of TMB. UV / Vis spectrum of 1,3,5-trimethoxybenzene TMB in acetonitrile at 20°C.

2 Results and Discussion

2.1 Quantitative evaluation of electrospray ionization mass spectra of A and AP

^{1}H-NMR spectroscopy is suitable to serve as a quantitative method only when the analytes are present in sufficiently high concentrations. To enable the determination of quantum yields at low concentration, high-resolution electrospray ionization mass spectrometry (HR-ESI MS) is employed here. HR-ESI MS is highly sensitive, but potential ionization biases prevent a straightforward quantification. Identical samples containing A and NEM as well as an internal standard (for NMR characterization) were prepared and irradiated with varied irradiation times, before being characterized by both ^{1}H-NMR spectroscopy and mass spectrometry. Conversion was determined via ^{1}H-NMR spectroscopy, allowing the development of a method to derive correct conversion values via quantitative evaluation of mass spectra.

Irradiation experiments with LED 3 were carried out with varied irradiation times using an irradiation setup as described previously.[1] A stock solution containing 1.0 mmol L^{-1} (1.0 eq.) α-methyl-ω-[(2-formyl-3-methylphenyl)thio]-poly(ethylene glycol) A, 1.15 mmol L^{-1} (1.15 eq.) *N*-ethylmaleimide NEM and 1.2 mmol L^{-1} (1.2 eq.) 1,3,5-Trimethoxybenzene TMB dissolved in CD_3CN was prepared. The stoichiometry was controlled via ^{1}H-NMR spectroscopy. For each sample, 0.5 mL of a stock solution (containing each 0.5 mmol A and 0.575 mmol NEM) was transferred to a glass vial containing a stir bar. The vial was crimped, and the solution deoxygenated by a stream of nitrogen for 10 min. The sample was fitted into the aluminium holder on top of LED 3[1] and the solution was stirred at 400 rpm (shortly before and during irradiation). Irradiation was conducted for the duration shown in Table S6.

Table S6: Irradiation times and conversion as determined by ^{1}H-NMR spectroscopy.

Irradiation time t / s	Conversion p / %
8	10.82
15	17.16
23	31.6
30	47.54
45	63.39
60	87
80	97.68
120	>99.5

For each sample shown in Table S6 the conversion is known. The samples were subjected to HR-ESI MS and the signals of double and triple charged species of A and AP were numerically integrated and apparent mole fractions determined (using a modified algorithm[2]). Apparent mole fractions are determined for each relevant pair of macromolecular species A and AP, which have the same number of ethylene glycol units in the backbone (number of repeating units), refer to Figure S13. For example, the integral of the principal ion of $A_{n=47}$ (47 ethylene glycol repeating units) and the integral of the principal ion of $AP_{n=47}$ are used to calculate the apparent mole fraction at this number of repeating units, refer to Figure S12 and Figure S13. For all measured samples, the apparent mole fraction is largely independent of the number of repeating units. Thus, in the following, apparent mole fraction refers to the average of these over the number of relevant repeating units (refer also to Figure S14). For each sample, the average of the apparent mole fraction of the double and triple charged species, multiplied with 1.05 is, with a small error (3%), equal to the conversion determined by ^{1}H-NMR spectroscopy (refer to Table S8).

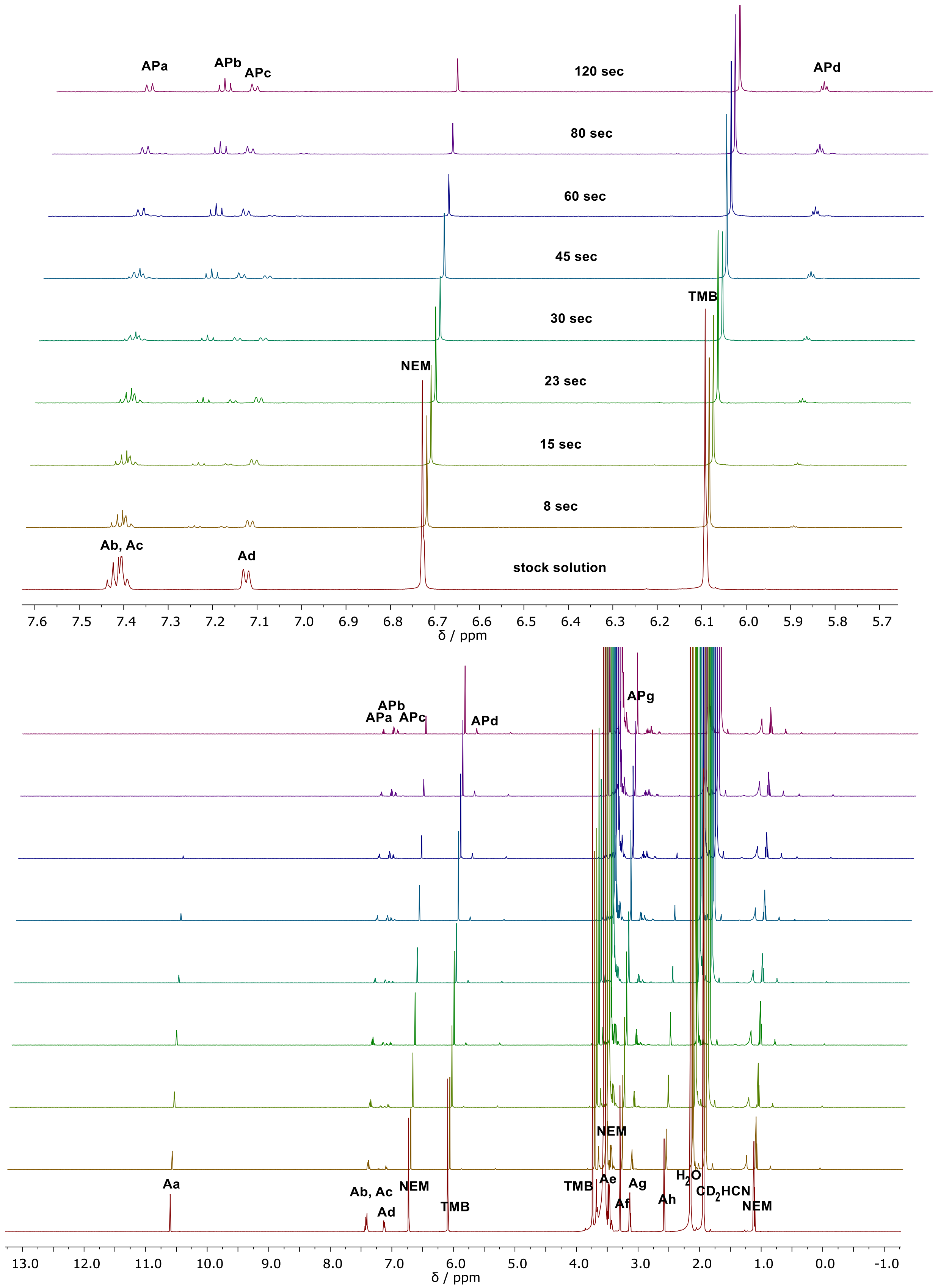

Figure S11: ^{1}H-NMR spectra of stock solution and irradiated samples. ^{1}H-NMR spectrum of a stock solution used for irradiation with LED 3 and the obtained spectra after irradiation.

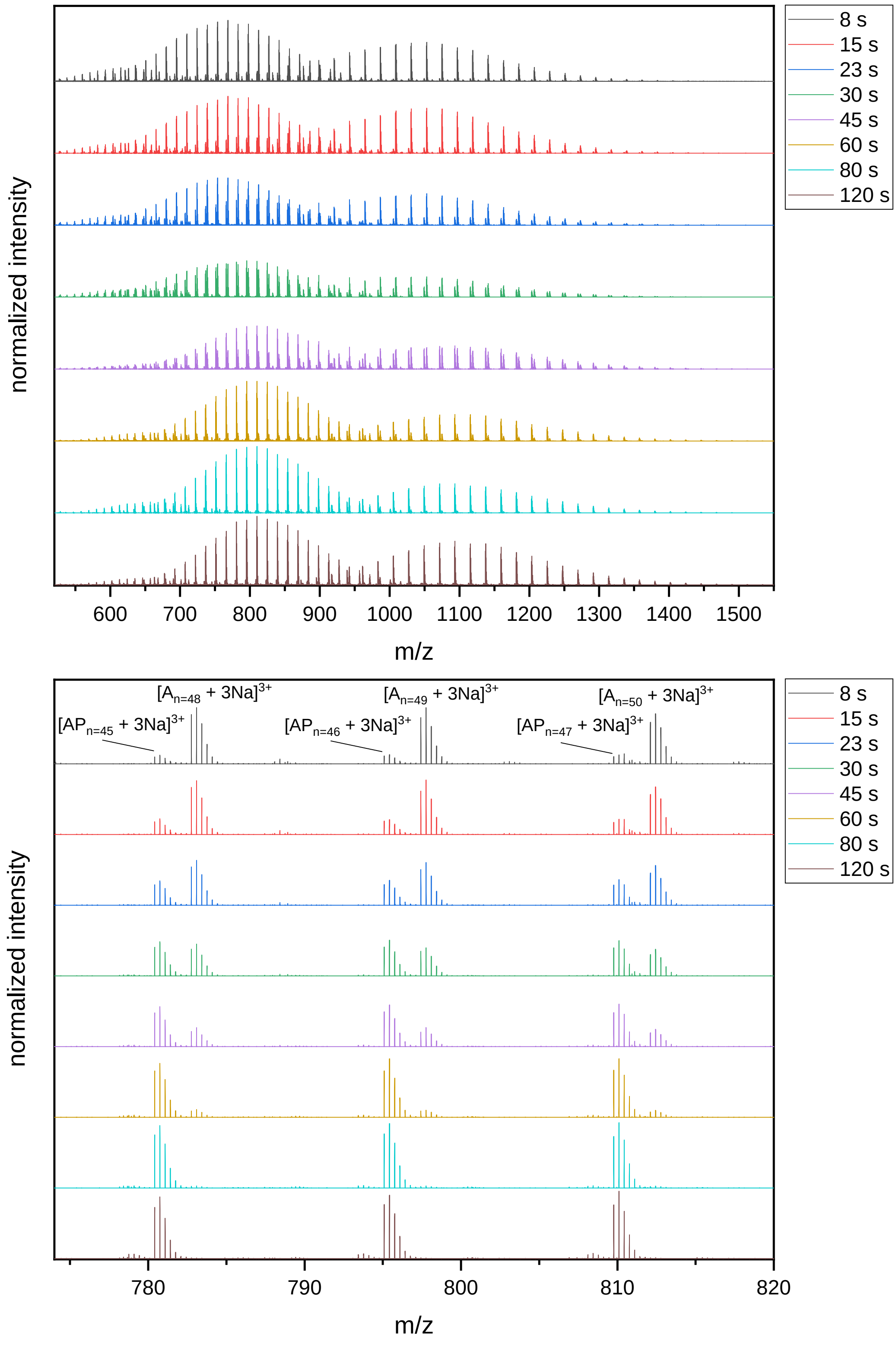

Figure S12: ESI-MS spectra of stock solution and irradiated samples. High-resolution electrospray ionization mass spectra of samples containing A, NEM and TMB, after irradiation with LED 3, refer also to Table S6. Above: Overview of the spectra showing the quadruple, triple and double charged species. Below: Expansion of the same spectra showing the triple charged region and assignment of relevant species, refer also to Table S7.

Table S7. Theoretical and experimentally observed m/z values of relevant species as shown in Figure S12. For clarity, only a selection of species from the top mass spectrum (sample with 8 seconds irradiation time) are listed here.

m/z_{exp}	assign-ment	ion assignment	formula	m/z_{theor}	Δm/z	R	$Δm/z_{max}$	deviation / ppm
782.7574	A	$[A_{n=48} + 3\ Na]^{3+}$	$C_{105}H_{202}O_{49}SNa_3$	782.7571	0.0003	150817	0.0052	0.38
780.4138	AP	$[AP_{n=45} + 3\ Na]^{3+}$	$C_{105}H_{197}NO_{48}SNa_3$	780.4134	0.0004	146429	0.0053	0.51

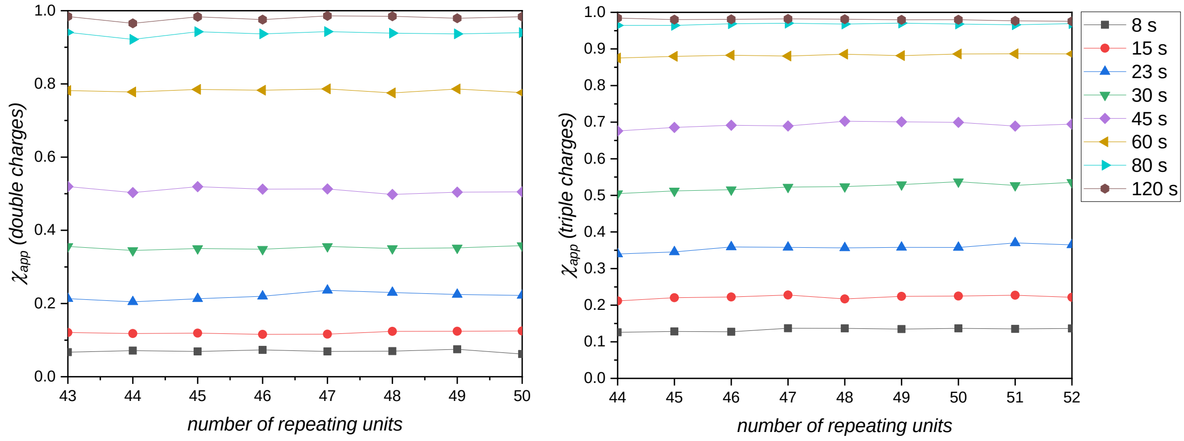

Figure S13: Apparent mole fractions determined from mass spectra. Apparent mole fractions as determined from mass spectra of the samples as shown in Table S6. Left: evaluation of double charged species. Right: evaluation of triple charged species.

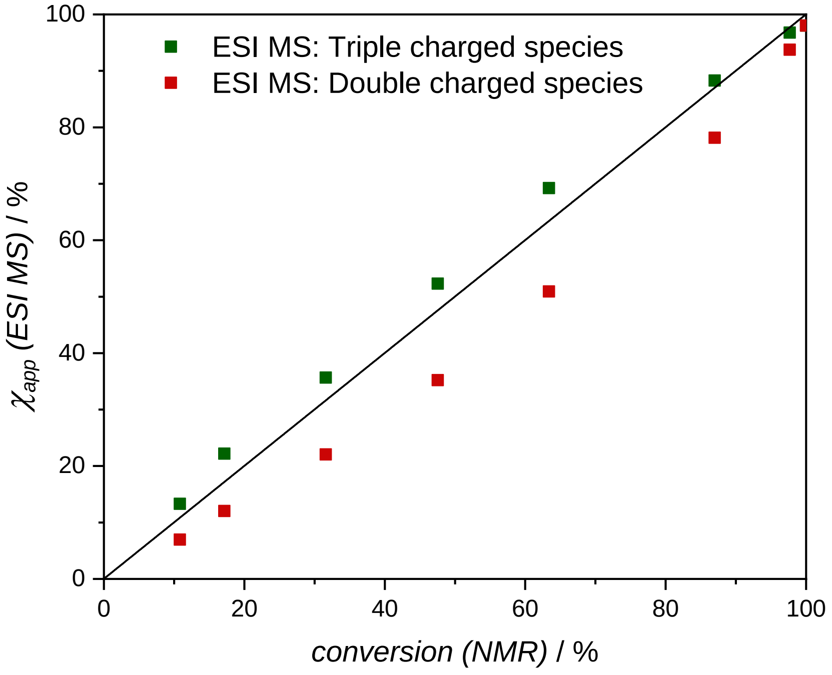

Figure S14: Comparison of mass spectrometric and ^{1}H-NMR spectroscopic response. Average apparent mole fractions of double and triple charged species found in each mass spectrum *vs.* conversion as determined by ^{1}H-NMR spectroscopy.

Table S8: Determination of correction factor cf for the quantitative evaluation of mass spectra of A and AP. The average of the correction factors cf reported below is 1.05, which is used for the determination of conversion by mass spectrometric measurement.

Irradiation time / s	χ_{app} (double charges)	χ_{app} (triple charges)	conversion (NMR) / %	cf
8	6.96	13.31	10.82	1.0676
15	12.04	22.19	17.16	1.0026
23	22.04	35.66	31.6	1.0953
30	35.2	52.32	47.54	1.0864
45	50.93	69.22	63.39	1.0552
60	78.14	88.28	87	1.0455
80	93.75	96.77	97.68	1.0254
120	98.03	98.01	100	1.0202

2.2 Quantum yields of photoenol ligation

2.2.1 Wavelength dependence

Quantum yields were calculated from the conversion of A to AP in a tunable laser experiment with known photon count. The sample solution was deoxygenated in a crimped 0.8 mL glass vial for 10 min by a stream of nitrogen. Each sample was subjected to monochromatic tunable laser irradiation according to the parameters listed in Table S3. Afterwards, each sample solution was transferred to an NMR tube and NMR spectroscopically characterized. In previous experiments it was found that the here employed deoxygenation procedure only to a very small degree reduces the amount of NEM by evaporation, while the amount of TMB remains unaffected. From the experimental parameters, the observed conversion and the molar attenuation coefficients of the light absorbing species, the respective quantum yields were determined iteratively, analogously to a previously published method.[2] The quantum yield at 382 nm was determined mass spectrometrically, as shown in section 2.6.

Table S9: Preparation of samples (the first row represents each sample prior to irradiation), irradiation with varied photon count $n_{p,\ total}$ and measured amounts of product and reactants. The amount of photons $n_{p,\ total}$ is calculated as the total number of photons during the experiment that reached the sample solution after passing through the bottom of the glass vial. The laser is operated at a repetition rate of 20 Hz (20 °C). The transmittance of the glass vials was previously determined (refer to Table S4). Conversion of A to the photoenol ligation product AP, calculated via the ratio of the integrals of A and AP is noted as $p_{A \rightarrow AP}$ (the quantum yield of the respective reaction as $\Phi_{A \rightarrow AP}$), whereas conversion, calculated via the decrease of the integrals of A vs the internal standard TMB is noted as $p_{A \rightarrow \Sigma APx}$ (the quantum yield of the respective reaction as $\Phi_{A \rightarrow \Sigma APx}$). Quantum yields were calculated using a previously established method of simulating tunable laser photoreactions with spatial and temporal precision.[2]

λ / nm	V_{CD3CN} / mL	$t_{irradiation}$ / s	E_{pulse} / µJ	$n_{p,\ total}$ / µmol	n_{TMB} / µmol	n_A / µmol	n_{AP} / µmol	n_{NEM} / µmol	$p_{A \rightarrow AP}$ / %	$p_{A \rightarrow \Sigma APx}$ / %	$\Phi_{A \rightarrow AP}$	$\Phi_{A \rightarrow \Sigma APx}$
-	0.25	0	0	0	0.64	0.5	0	0.63	0	0	-	-
285	0.25	160	396	0.844	0.64	0.42	0.050	0.54	10.6	15.5	0.050	0.076
292	0.25	200	211	0.874	0.5	0.43	0.063	0.52	12.9	14.5	0.08	0.089
300	0.25	71	396	0.822	0.64	0.43	0.060	0.54	12.4	15.0	0.10	0.122
307	0.25	120	197	0.820	0.5	0.43	0.062	0.52	12.6	14.5	0.089	0.103
315	0.25	54	396	0.827	0.64	0.40	0.085	0.53	17.4	19.5	0.10	0.116
322	0.25	101	197	0.819	0.5	0.43	0.067	0.54	13.4	13.5	0.066	0.067
330	0.25	48	400	0.834	0.64	0.42	0.062	0.55	12.7	15.5	0.054	0.065
337	0.25	92	198	0.823	0.5	0.44	0.045	0.56	9.3	12.5	0.035	0.048
345	0.25	44	401	0.828	0.64	0.45	0.038	0.58	7.8	10.0	0.029	0.035
360	0.25	42	398	0.836	0.64	0.46	0.042	0.59	8.3	8.5	0.027	0.028
375	0.25	79	203	0.847	0.5	0.46	0.037	0.59	7.3	7.5	0.022	0.024
382	0.25	180	104	0.995	0.5	0.45	0.049	-	9.7	-	0.027	-
390	0.25	38	760	0.848	0.64	0.46	0.038	0.60	7.7	7.5	0.029	0.028
397	0.25	49	306	0.851	0.64	0.47	0.027	0.60	5.4	6.5	0.027	0.031
405	0.25	780	238	10.8	0.5	0.37	0.100	0.51	21.2	25.5	0.015	0.019
412	0.25	220	1360	17.7	0.5	0.46	0.035	0.58	7.1	8.0	0.0053	0.006
420	0.25	550	1530	50.8	0.5	0.48	0.015	0.61	3.0	4.5	0.0015	0.0024
427	0.25	300	2360	43.4	0.5	0.50	0.0	0.63	0.0	0.0	0.00	0.00

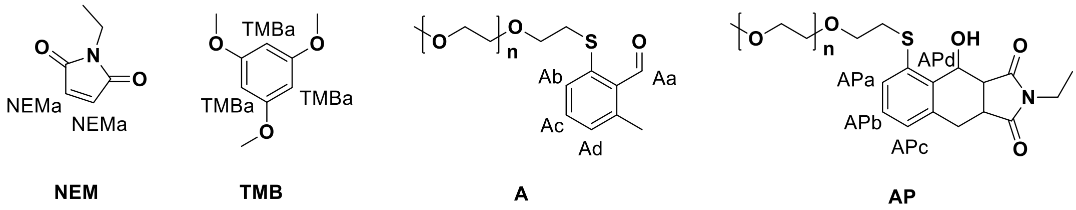

Figure S15: Structures and assignment for the spectra shown in Figure S16.

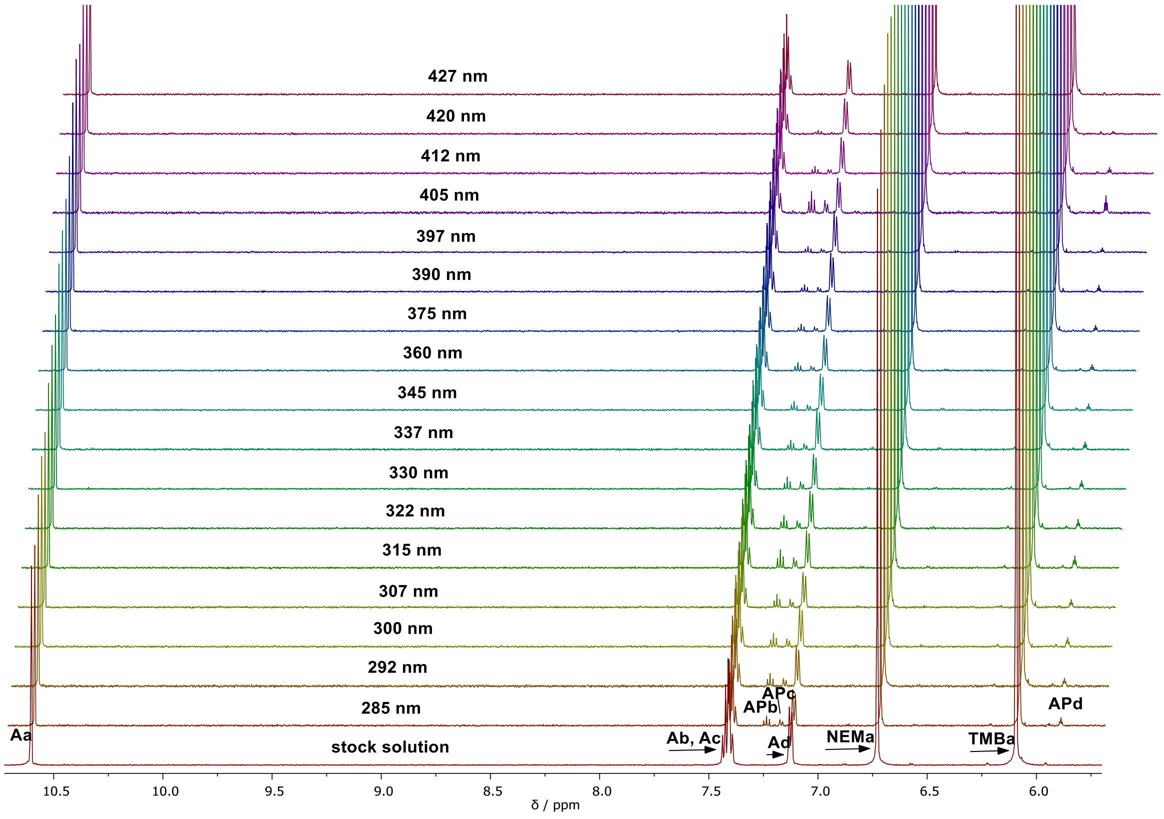

Figure S16: ^{1}H-NMR spectra. ^{1}H-NMR spectra of a stock solution and samples that were used to determine conversion and quantum yields of photoenol ligation in acetonitrile (refer to Table S9). Conversion and amounts of each compound were determined by integration of signals Aa, Ad, APb, APc, APd, NEMa and TMBa, refer also to Figure S15.

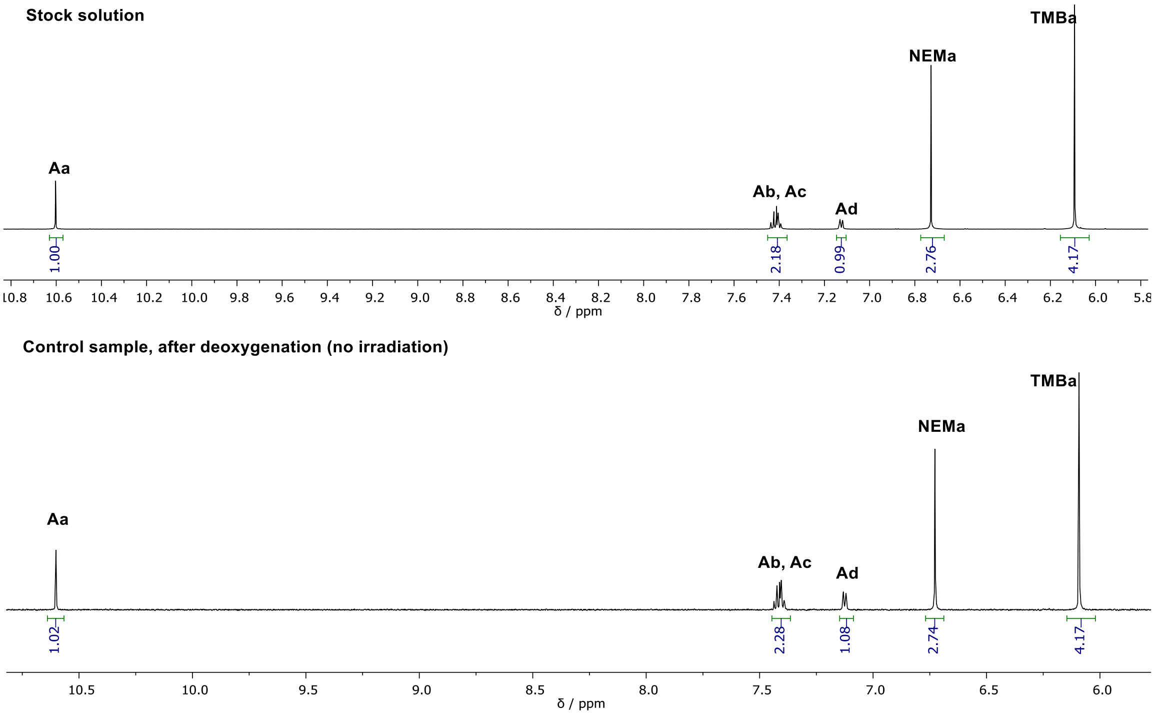

Figure S17: ^{1}H-NMR spectra. ^{1}H-NMR spectra of the stock solution used for the wavelength-dependent irradiation experiments and respective control samples, which was drawn from the same stock solution, deoxygenated by a stream of nitrogen, but not irradiated.

The above-described experiments and calculations for the determination of wavelength-dependent quantum yields were repeated two more times for each wavelength in the range of 285 nm to 420 nm, see below. The conversion at 382 nm was determined in these cases ^{1}H-NMR spectroscopically. The average of the three quantum yields for each wavelength and respective standard deviation are displayed in Figure 2b in the main publication. An additional control sample was prepared from the same stock solution and this control sample was not irradiated. The ratios of **A**, **NEM** and **TMB** are, within the error of the NMR measurement equal after deoxygenation, see Figure S17.

Table S10: Preparation of samples (the first row represents each sample prior to irradiation), irradiation with varied photon count $n_{p,\,total}$ and measured amounts of product and reactants. The amount of photons $n_{p,\,total}$ is calculated as the total number of photons during the experiment that reached the sample solution after passing through the bottom of the glass vial. The laser is operated at a repetition rate of 20 Hz (20 °C). The transmittance of the glass vials was previously determined (refer to Table S4). Conversion of **A** to the photoenol ligation product **AP,** calculated via the ratio of the integrals of **A** and **AP** is noted as $p_{A\rightarrow AP}$ (the quantum yield of the respective reaction as $\Phi_{A\rightarrow AP}$), whereas conversion, calculated via the decrease of the integrals of **A** vs the internal standard **TMB** is noted as $p_{A\rightarrow \Sigma APx}$ (the quantum yield of the respective reaction as $\Phi_{A\rightarrow \Sigma APx}$). Quantum yields were calculated using a previously established method of simulating tunable laser photoreactions with spatial and temporal precision.[2]

λ / nm	V_{CD3CN} / mL	$t_{irradiation}$ / s	E_{pulse} / µJ	$n_{p,\,total}$ / µmol	n_{TMB} / µmol	n_A / µmol	n_{AP} / µmol	n_{NEM} / µmol	$p_{A\rightarrow AP}$ / %	$p_{A\rightarrow \Sigma APx}$ / %	$\Phi_{A\rightarrow AP}$	$\Phi_{A\rightarrow \Sigma APx}$
285	0.25	360	182	0.873	0.68	0.43	0.053	0.56	11.1	14.5	0.053	0.072
285	0.25	360	183	0.878	0.62	0.41	0.045	0.55	9.9	18.0	0.046	0.093
292	0.25	300	140	0.870	0.68	0.44	0.057	0.56	11.5	12.5	0.074	0.081
292	0.25	242	184	0.922	0.62	0.41	0.060	0.52	12.7	17.5	0.078	0.112
300	0.25	300	92	0.806	0.68	0.42	0.063	0.55	13.1	16.0	0.116	0.146
300	0.25	180	140	0.736	0.62	0.42	0.055	0.55	11.5	15.5	0.110	0.153
307	0.25	120	220	0.916	0.68	0.40	0.087	0.55	17.7	19.5	0.125	0.139
307	0.25	120	230	0.957	0.62	0.39	0.092	0.51	19.2	23.0	0.131	0.161
315	0.25	70	327	0.885	0.68	0.43	0.073	0.59	14.6	14.5	0.083	0.083

315	0.25	70	309	0.837	0.62	0.38	0.085	0.52	18.2	23.5	0.112	0.150
322	0.25	100	209	0.860	0.68	0.41	0.075	0.56	15.5	18.0	0.077	0.091
322	0.25	60	351	0.866	0.62	0.38	0.102	0.51	21.3	25.0	0.109	0.131
330	0.25	60	195	0.508	0.68	0.47	0.032	0.63	6.3	6.5	0.043	0.044
330	0.25	60	253	0.659	0.62	0.45	0.048	0.59	9.7	10.5	0.052	0.056
337	0.25	100	185	0.835	0.68	0.44	0.053	0.59	10.8	12.0	0.041	0.046
337	0.25	60	282	0.764	0.62	0.44	0.042	0.59	8.7	12.5	0.036	0.052
345	0.25	100	224	1.052	0.68	0.44	0.052	0.62	10.5	12.0	0.029	0.034
345	0.25	80	292	1.097	0.62	0.43	0.060	0.58	12.3	14.5	0.033	0.040
360	0.25	100	214	1.070	0.68	0.44	0.057	0.59	11.5	13.0	0.029	0.034
360	0.25	90	244	1.098	0.62	0.43	0.048	0.58	10.1	14.0	0.025	0.035
375	0.25	100	216	1.141	0.68	0.41	0.075	0.59	15.5	18.0	0.038	0.045
375	0.25	90	230	1.093	0.62	0.43	0.045	0.57	9.6	15.0	0.024	0.038
382	0.25	120	143	0.928	0.68	0.46	0.052	0.63	10.2	9.0	0.031	0.027
382	0.25	120	148	0.961	0.62	0.44	0.043	0.57	9.0	12.5	0.026	0.037
390	0.25	150	179	1.491	0.68	0.43	0.073	0.59	14.6	14.5	0.032	0.032
390	0.25	140	191	1.485	0.62	0.43	0.057	0.57	11.6	14.0	0.025	0.031
397	0.25	120	124	0.844	0.68	0.47	0.023	0.63	4.7	5.5	0.023	0.026
397	0.25	100	142	0.806	0.62	0.46	0.020	0.61	4.1	7.5	0.021	0.038
405	0.25	1800	90	9.41	0.68	0.40	0.115	0.56	22.4	20.5	0.019	0.017
405	0.25	1200	75	5.23	0.62	0.42	0.060	0.58	12.6	16.5	0.018	0.024
412	0.25	1200	248	17.62	0.68	0.46	0.062	0.62	11.8	8.0	0.009	0.006
412	0.25	180	1340	14.28	0.62	0.45	0.052	0.60	10.2	9.5	0.0097	0.009
420	0.25	1800	305	33.15	0.68	0.50	0.018	0.66	3.6	0.5	0.003	0.0005
420	0.25	600	1550	56.16	0.62	0.47	0.033	0.61	6.7	7.0	0.0033	0.0035

2.2.2 Concentration dependence

To enable the determination of the conversion of an experiment with significantly lower amounts of compounds (lower concentration), high-resolution electrospray ionization mass spectrometry and the evaluation method as described in section 2.1 was employed for the samples described here. Sample solutions for tunable laser experiments were prepared analogously to the previous tunable laser experiments, but with the concentrations and other parameters as shown in Table S10. Loss of acetonitrile during deoxygenation with nitrogen gas was quantified in the following way and is accounted for in Table S10. For each experiment, either 0.125 mL, 0.25 mL or 0.5 mL acetonitrile were used. Three vials containing only acetonitrile (0.125 mL, 0.25 mL and 0.5 mL), respectively, were weighed, deoxygenated with nitrogen gas for 10 minutes and weighed again to determine the amount of evaporated solvent. In case of 0.125 mL (0.25 mL, 0.5 mL) acetonitrile before deoxygenation, 17 mg (22 mg, 24 mg) mass loss were observed. The volume of acetonitrile and concentration in Table S11 (V_{CD3CN} before deoxygenation is 0.125, 0.25 or 0.5 mL) are corrected, respectively.

Table S11: Irradiation times and conversion as determined by high-resolution mass spectrometry. All experiments were conducted at the wavelength 382 nm. The sample, which is not irradiated, shows that the formation of the product **AP** is negligible in this case, see also Figure S8.

c / mol L^{-1}	V_{CD3CN} / mL	$t_{irradiation}$ / s	E_{pulse} / μJ	$n_{p, total}$ / μmol	$p_{A\rightarrow AP}$ / %	$\Phi_{A\rightarrow AP}$
4.8 * 10^{-3}	0.103	360	105	2.01	11.2	0.016
2.3 * 10^{-3}	0.22	180	104	0.99	9.7	0.027
1.1 * 10^{-3}	0.47	120	106	0.68	9.2	0.038
5.3 * 10^{-4}	0.47	90	106	0.51	12.8	0.045
1.3 * 10^{-4}	0.47	40	101	0.21	11.0	0.057
6.7 * 10^{-5}	0.47	0	-	0.0	0.04	-

To investigate the observed trend at a different wavelength, three experiments at varied initial concentration at 307 nm were carried out and conversion was quantified with NMR spectroscopy, see Table S12 and Figure S18.

Table S12: Irradiation times and conversion as determined by NMR spectroscopy. All experiments were conducted at the wavelength 307 nm.

c / mol L^{-1}	V_{CD3CN} / mL	$t_{irradiation}$ / s	E_{pulse} / µJ	$n_{p, total}$ / µmol	$p_{A\rightarrow AP}$ / %	$\Phi_{A\rightarrow AP}$
4.8 * 10^{-3}	0.103	180	107	0.67	6.5	0.059
2.3 * 10^{-3}	0.22	180	108	0.67	11.3	0.103
1.1 * 10^{-3}	0.22	120	106	0.44	17.8	0.133

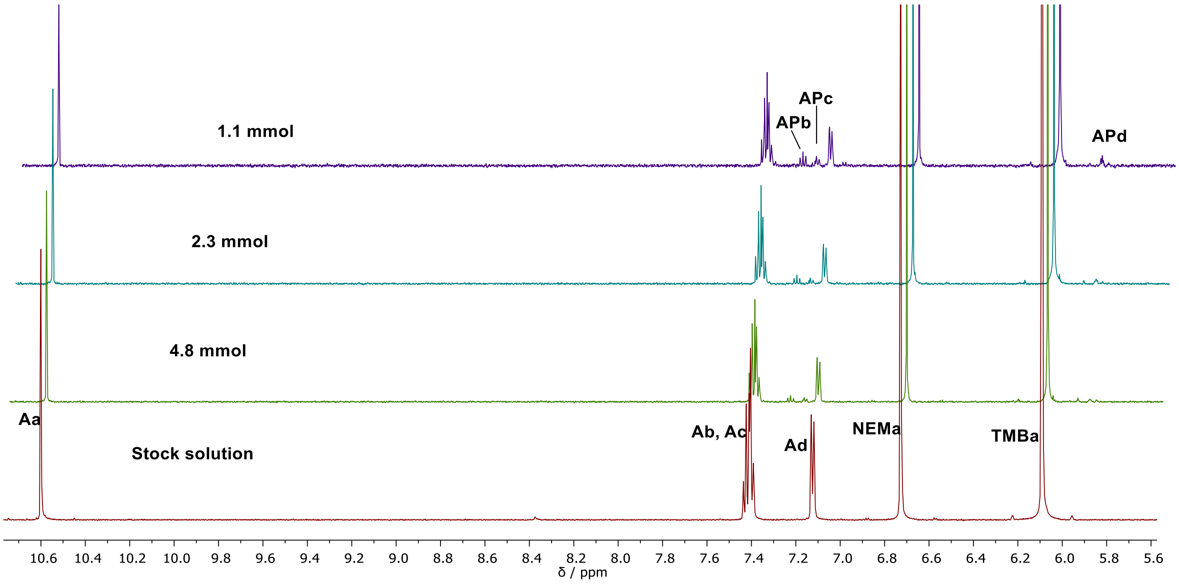

Figure S18: ^{1}H-NMR spectra. ^{1}H-NMR spectra of samples irradiated at 307 nm with varied initial concentration, see Table S12.

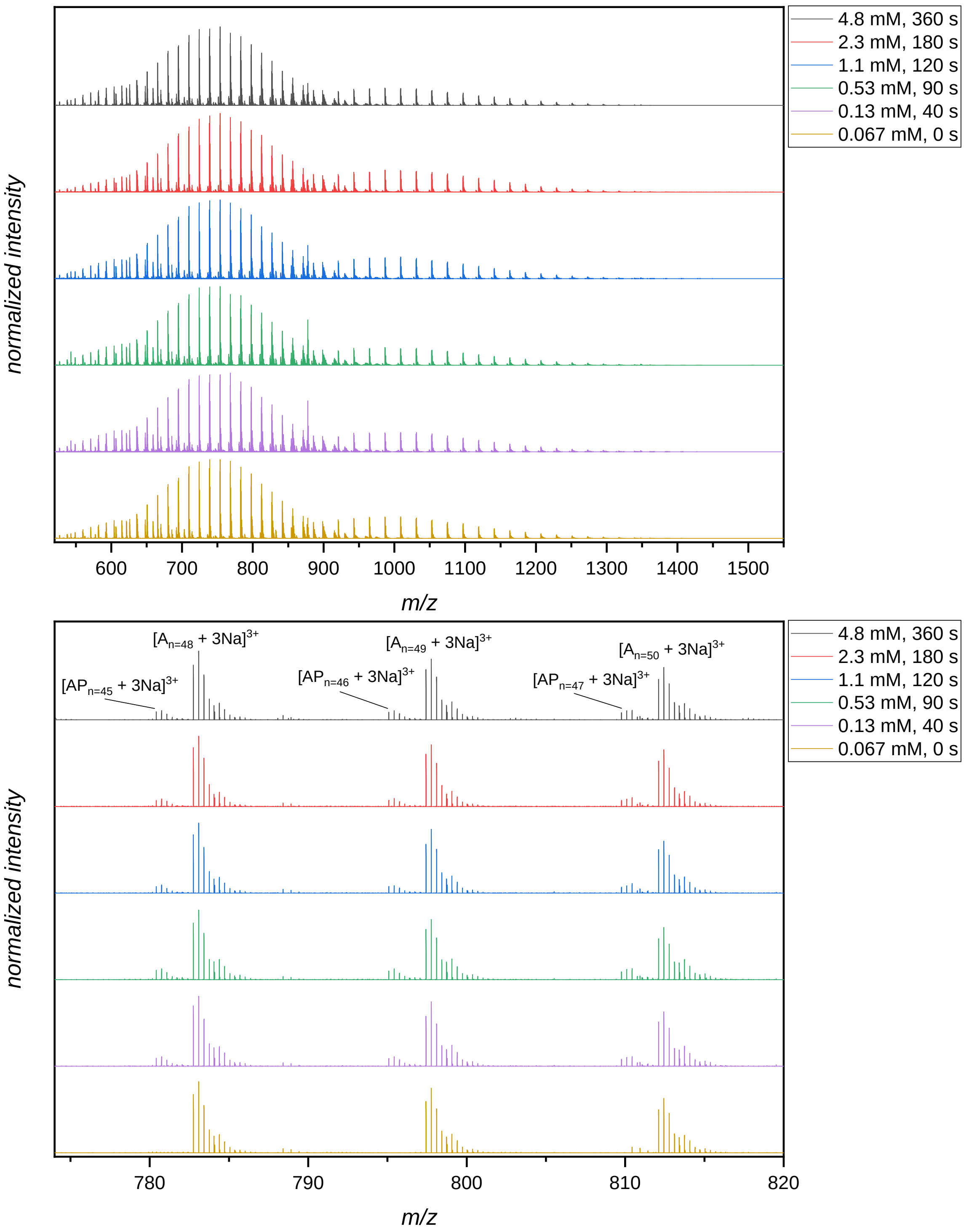

Figure S19: Mass spectra of the samples irradiated according to Table S11.

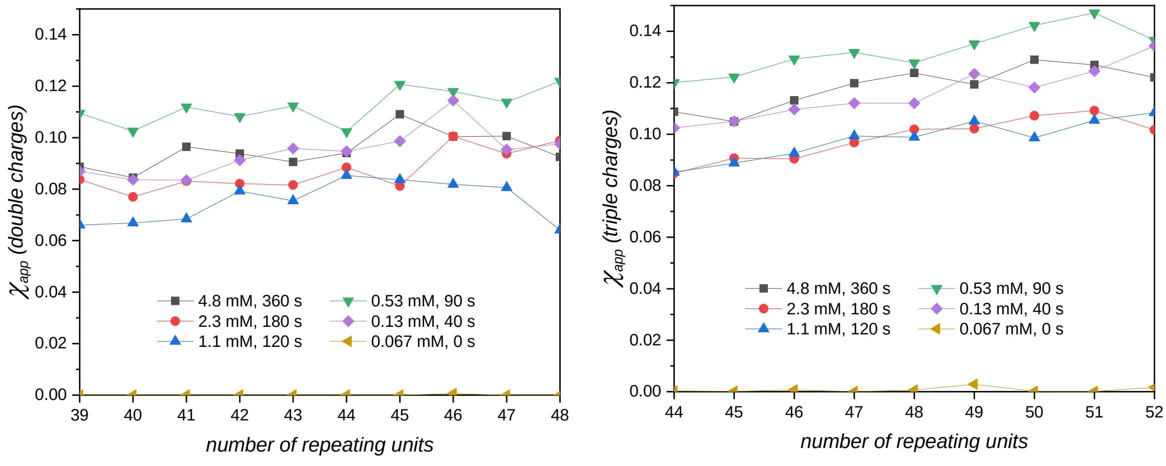

Figure S20: Apparent mole fractions of AP in samples irradiated according to Table S11.

2.3 Curve fitting for wavelength and concentration dependent quantum yields

The numerical simulation requires an analytical expression of the quantum yield in dependence on both wavelength and concentration. Thus, a curve was fitted to both the wavelength and concentration dependent quantum yield data, see Figure S21 and equation [5] and [6], respectively. The two expressions are combined to an expression dependent on both wavelength and concentration, see Figure S22 and equation [7].

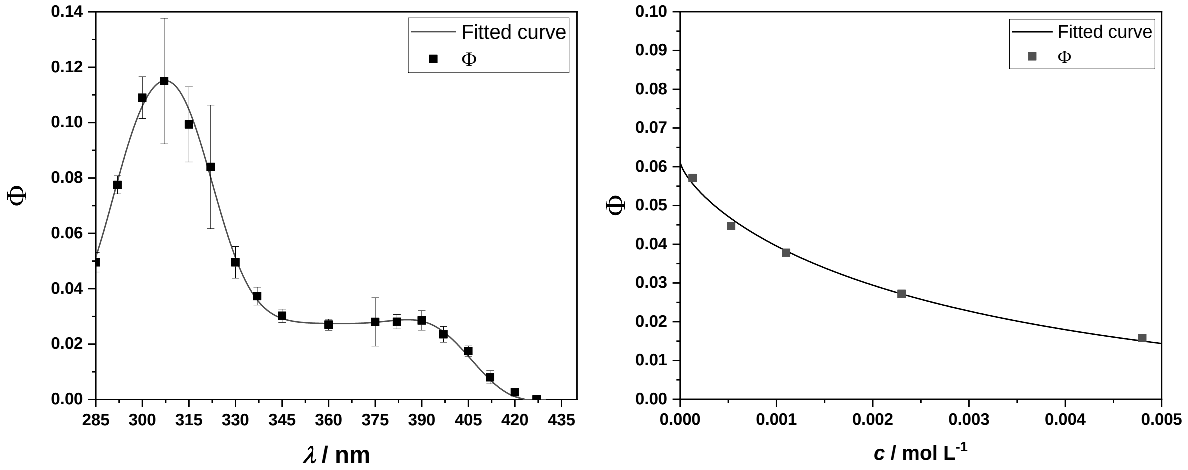

Figure S21: Fitted curves for quantum yields. Left: Wavelength dependent quantum yields and respective fitted curve, see also equation [5]. The values are averages of three experiments and the error bars show the standard deviation of these values. Right: Concentration dependent quantum yields and respective fitted curve, see also equation [6].

$$\boldsymbol{\Phi}_{\lambda,\,2\,mM} = \frac{\sqrt{(\lambda-r)^2}-(\lambda-r)}{2*(r-\lambda)} * \left(k + l\,e^{-\frac{(\lambda-m)^2}{2\,n^2}} + \,o\,e^{-\frac{(\lambda-p)^2}{2\,q^2}} - w\,e^{-\frac{(\lambda-r)^2}{2\,s^2}} + t\,e^{-\frac{(\lambda-u)^2}{2\,v^2}}\right) \quad [5]$$

Table S13: Parameters used in equation [5] for the fitted curve shown in Figure S21.

Parameter	Values for [5]
r	423
k	0.0275
l	0.08
m	310.5
n	12.5
o	0.026
p	295
q	9
s	20
t	0.009
u	396
v	12
w	0.028

$$\boldsymbol{\Phi}_{382\,nm,\,\boldsymbol{c}} = 0.0612 * e^{-75*\boldsymbol{c}^{0.745}} \qquad [6]$$

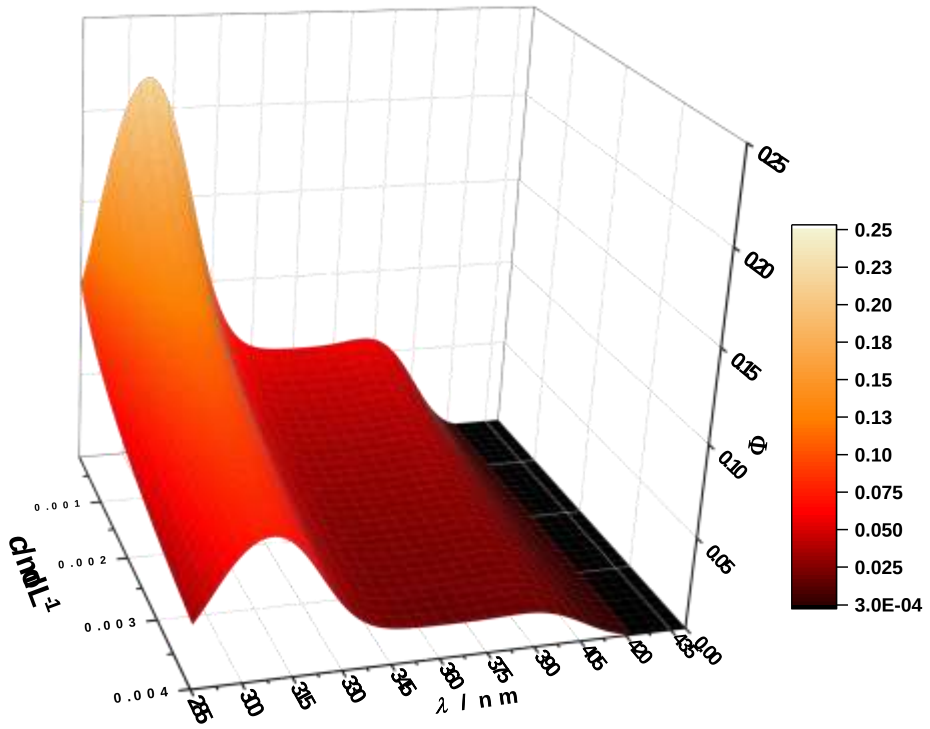

Figure S22: Wavelength and concentration dependent quantum yields. Wavelength and concentration dependent quantum yields according to equation [7], see also Table S11.

In equation [7], the value 2.25 is calculated from $\Phi_{382\text{ nm, 0 mM}}$ = 0.0612 divided by $\Phi_{382\text{nm, 2.3 mM}}$ = 0.0272, because $\Phi_{382\text{nm, 2.3 mM}}$ is the intersection of equation [5] and [6].

$$\boldsymbol{\Phi}_{\boldsymbol{\lambda},\,\boldsymbol{c}} = \frac{\sqrt{(\boldsymbol{\lambda}-r)^2}-(\boldsymbol{\lambda}-r)}{2*(r-\boldsymbol{\lambda})} * 2.25 * \left(e^{-20.5*\boldsymbol{c}^{0.505}} * l\, e^{-\frac{(\lambda-m)^2}{2\,n^2}} + \left(k + o\, e^{-\frac{(\lambda-p)^2}{2\,q^2}} - w\, e^{-\frac{(\lambda-r)^2}{2\,s^2}} + t\, e^{-\frac{(\lambda-u)^2}{2\,v^2}}\right) * e^{-75*\boldsymbol{c}^{0.745}}\right) \qquad [7]$$

Equation [8] is a mathematical expression (interpolation) for the wavelength-dependent quantum yields of the reaction of **B** with **NEM** to **BP**, as reported previously.[2] The expression and the original values are shown in Figure S23.

$$\boldsymbol{\Phi}_{\boldsymbol{\lambda},\,\boldsymbol{c}} = \frac{\sqrt{(\boldsymbol{\lambda}-t)^2}-(\boldsymbol{\lambda}-t)}{2*(t-\boldsymbol{\lambda})} * \left(a\,e^{-\frac{(\lambda-b)^2}{2\,c^2}} + d\,e^{-\frac{(\lambda-f)^2}{2\,g^2}} + h\,e^{-\frac{(\lambda-i)^2}{2\,j^2}} + k\,e^{-\frac{(\lambda-l)^2}{2\,m^2}} + n\,e^{-\frac{(\lambda-o)^2}{2\,p^2}} + q\,e^{-\frac{(\lambda-r)^2}{2\,s^2}}\right) \quad [8]$$

Table S14: Parameters used in equation [8] for the fitted curve shown in Figure S23.

Parameter	Values for [8]
a	0.35
b	288
c	21
e	0.78
f	321
g	30
h	0.29
i	347
j	13
k	0.08
l	360
m	10
n	0.17
o	377
p	12
q	-0.045
r	400
s	5
t	400.99

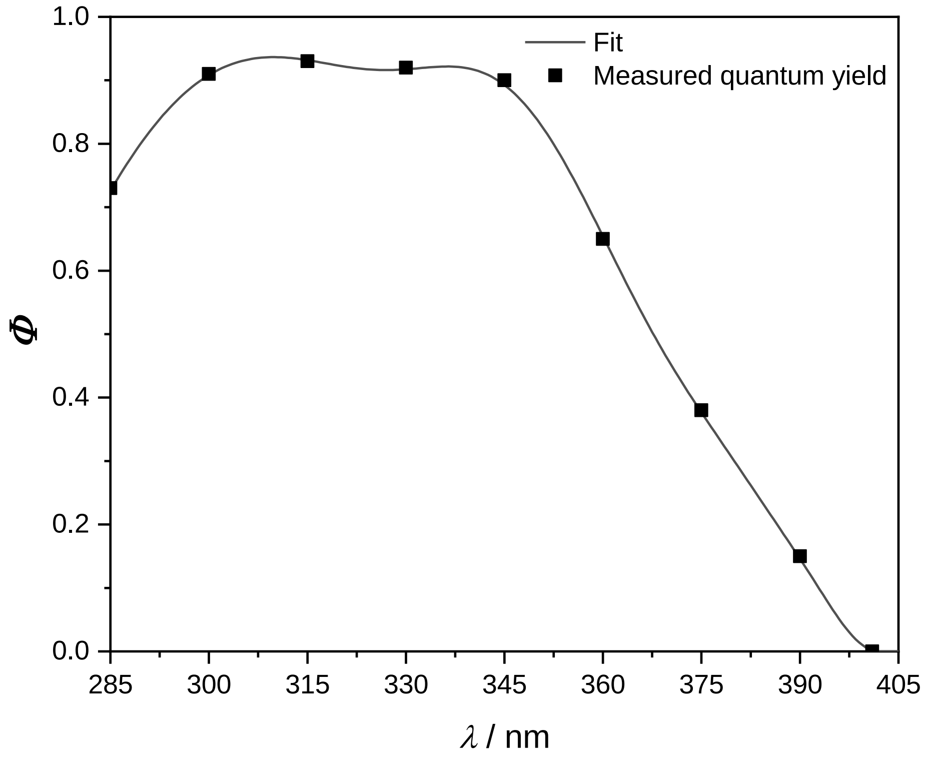

Figure S23: Wavelength-dependent quantum yields of the reaction of B with NEM to BP and fitted curve.

2.4 Evidence for intramolecular noncovalent interactions between oxygen and sulfur in A causing conformers and their influence on wavelength-dependent quantum yields

In the following, data supporting a comprehensive interpretation of the observed changes in absorbance and quantum yields depending on the substituents of *o*-methylbenzaldehydes and the wavelength is shown.

2.4.1 Comparison of UV Vis spectra of relevant derivatives of A and solvent dependence of absorbance

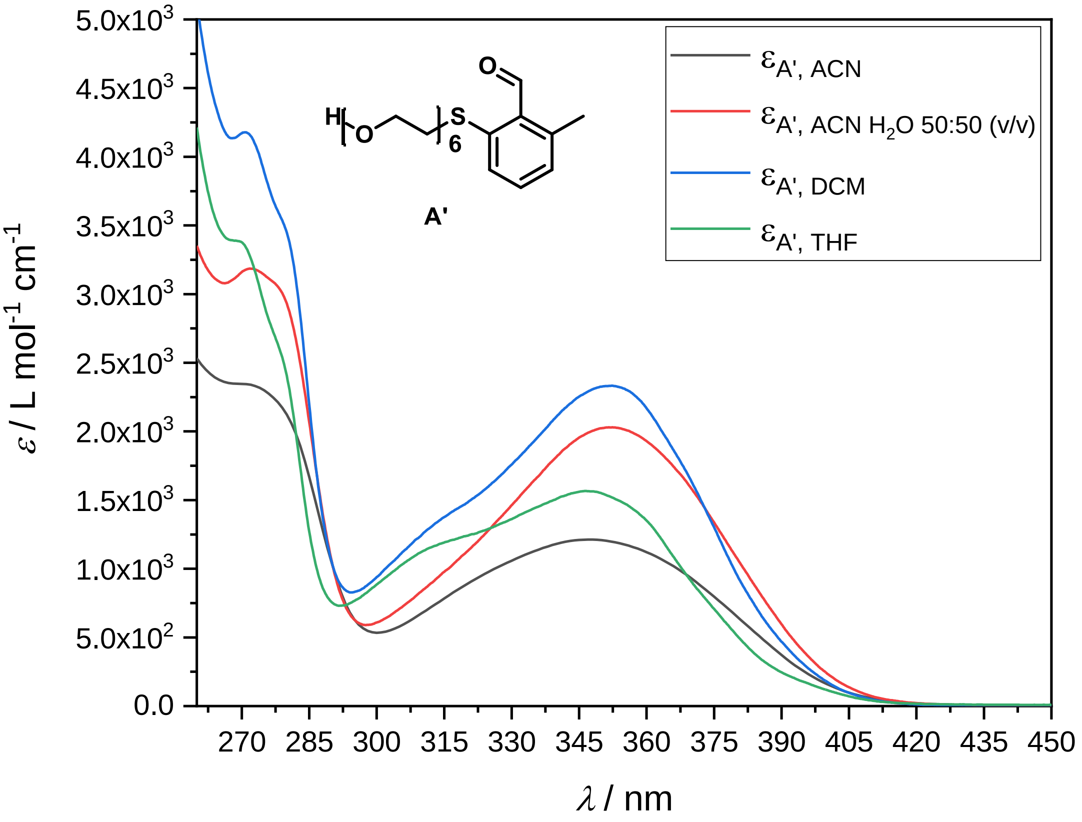

Figure S24: UV/Vis spectra of A' in varied solvents. UV/Vis spectra of 2-((17-hydroxy-3,6,9,12,15-pentaoxaheptadecyl)thio)-6-methylbenzaldehyde A' measured at 20°C in acetonitrile, acetonitrile – water (50:50, v/v), dichloromethane and tetrahydrofuran.

2.4.2 Wavefunction and density functional theory calculations

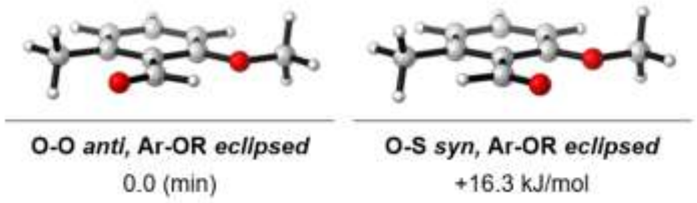

Figure S25: Relative Conformational Energies of EtherOMB.
Calculated with DLPNO-CCSD(T)/cc-pVTZ//B3LYP-D3BJ/def2-TZVP and SMD solvent corrections.

Table S15: Ether O-O *anti,* Ar-OR *eclipsed*

State (Character)	Excitation Energy/ eV	Wavelength/ nm	Oscillator Intensity
S_1 (nπ*)	3.62	342	0.00005
S_2 (ππ*)	3.87	320	0.11493
T_1(ππ*)	3.16	-	-
T_2(nπ*)	3.31	-	-
T_3(ππ*)	3.70	-	-

- The S_1 state possesses nπ* character at the Frank Condon geometry, with clear energetic separation of the S1/S2 states (0.25 eV). No fluorescence anticipated.
- Appropriate S_1/T_3 energy gap (0.08 eV) for El-Sayed allowed $(n\pi^*)^1/(\pi\pi^*)^3$ ISC.
- The T_1 state possesses ππ* character at the Frank Condon geometry but the nπ* T_2 state is energetically accessible (0.15 eV). This gap drops to 0.05 eV if an approximate $(n\pi^*)^3$ DFT geometry is used instead of the optimised S_0 DFT geometry. Allowing for equilibrium solvation (considering the slow response term) would also likely lower this further.

Table S16: ThioEther O-S *anti,* Ar-SR *eclipsed*

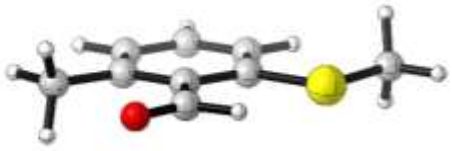

State (Character)	Excitation Energy/ eV	Wavelength/ nm	Oscillator Intensity
S_1 (nπ*)	3.60	345	< 0.00001
S_2 (ππ*)	3.67	338	0.09558
S_3 (ππ*)	4.77	260	0.11915
T_1(ππ*)	3.07	-	-
T_2(nπ*)	3.29	-	-
T_3(ππ*)	3.65	-	-

- The S_1/S_2 states are predicted to be essentially degenerate at the Frank Condon geometry, so fluorescence from the ππ* S_2 could outcompete ISC from nπ* S_1.
- Appropriate S_1/S_3 energy gap (0.05 eV) for El-Sayed allowed $(n\pi^*)^1/(\pi\pi^*)^3$ ISC.
- The T_1 state possesses ππ* character at the Frank Condon geometry but the nπ* T_2 state is energetically accessible (0.22 eV).

Table S17: ThioEther O-S *syn,* Ar-SR *eclipsed*

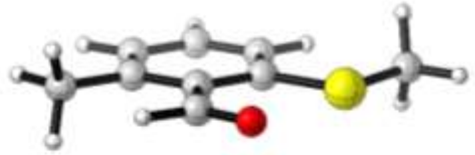

State (Character)	Excitation Energy/ eV	Wavelength/ Nm	Oscillator Intensity
S_1 (nπ*)	3.61	343	0.00003
S_2 (ππ*)	3.68	337	0.11703
S_3 (ππ*)	4.75	261	0.11019
T_1(ππ*)	3.06	-	-
T_2(nπ*)	3.28	-	-
T_3(ππ*)	3.61	-	-

- The S_1/S_2 states are predicted to be essentially degenerate at the Frank Condon geometry, so fluorescence from the ππ* S_2 could outcompete ISC from nπ* S_1.
- Appropriate S_1/S_3 energy gap (0 eV) for El-Sayed allowed $(n\pi^*)^1/(\pi\pi^*)^3$ ISC.
- The T_1 state possesses ππ* character at the Frank Condon geometry but the nπ* T_2 state is energetically accessible (0.22 eV).

Table S18: ThioEther O-S *anti,* Ar-SR *staggered*

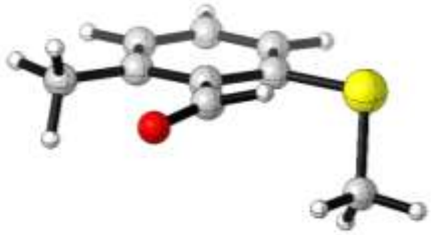

State (Character)	Excitation Energy/ eV	Wavelength/ nm	Oscillator Intensity
S_1 (nπ*/ππ*)	3.57	347	0.00769
S_2 (nπ*/ππ*)	4.16	298	0.05677
S_3 (nπ*/ππ*)	4.72	263	0.04158
T_1(nπ*/ππ*)	3.20	-	-
T_2(nπ*/ππ*)	3.31	-	-
T_3(nπ*/ππ*)	3.91	-	-

- Relatively bright S_1 state through mixing of nπ*/ππ* orbitals, fluorescence anticipated.
- Unclear application of El-Sayed selection rules because of mixed orbital character. Significant energy gap between S_1 and T_2 (0.26 eV) at Frank Condon geometry.
- Likely poor H-abstraction kinetics because of mixed nπ*/ππ* character of T_1.

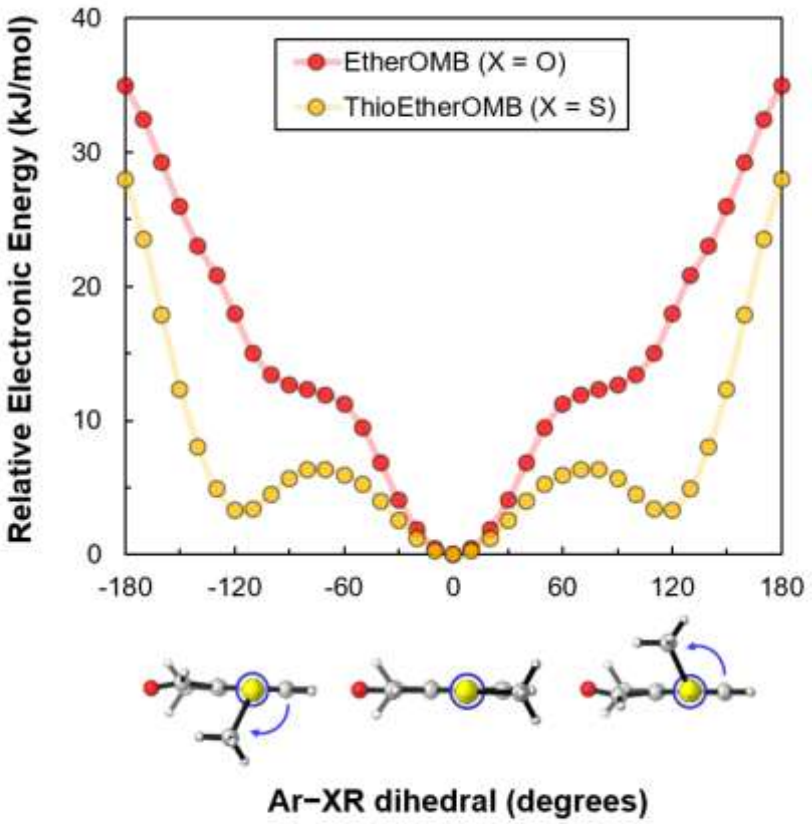

Figure S26: Relative electronic energy dependent on dihedral angle. B3LYP-D3BJ/def2-TZVP relaxed potential energy scans around the Ar-XR dihedral of the anti-conformations of EtherOMB (red) and ThioEtherOMB (yellow).

2.5 Simulation of LED light induced photoreactions and prediction of conversion

A simulation was previously introduced by us, which allows the determination of quantum yields from monochromatic tunable laser experiments.[2] The context in which the previously used algorithm (numerical simulation, monochromatic) is employed in this work as well as how the here introduced algorithm (numerical simulation, polychromatic) is applied is shown in Figure S27.

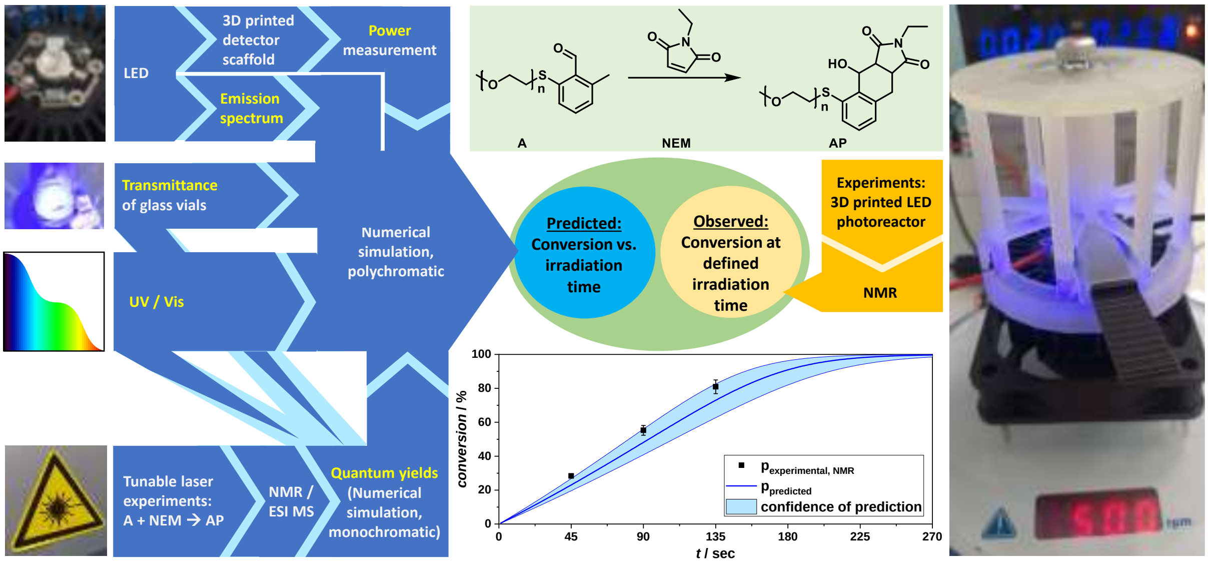

Figure S27: Overview of the approach for prediction of photochemical conversion. Overview of the approach taken here to quantitatively predict conversion of LED light induced photoreactions of thioether o-methylbenzaldehydes A with N-ethylmaleimide NEM. A 3D printed LED batch precision photoreactor (depicted on the right) and a respective 3D printed LED detector scaffold allow photoreactions with LEDs to be performed reproducibly with a measurable incident light intensity. The latter in combination with the emission spectrum of the LED, the transmittance of the sample glass vials, the absorbance of the involved chromophores and the (wavelength and concentration dependent) quantum yields are entering a numerical simulation, which quantitatively predicts the photoreaction outcome.

The progress of the photoreactions is simulated using modified source code written in the programming language python (python 3).[1,2,6,7]
At the core of the algorithm is the following concept:
The Beer Lambert law can be applied to homogenous solutions, describing the attenuation of light according to the concentration of light absorbing chromophores. The law allows to calculate the fraction of photons absorbed by the various chromophores depending on their molar attenuation coefficient. Thus, with a known number of photons (monochromatic light) entering a solution with known quantity of well-characterized chromophores it can be calculated how many photons are delivered into each respective chromophore and how many photons pass through the solution. The reaction quantum yield of each activatable ligation substrate then predicts the amount of respective product molecules formed.
Before predicting reaction progress, the initial state of the simulation is calculated as entries in lists. These contain information about the amount of each species in each segment. Due to the dimensions of the glass vial, the diameter of a segment is equal to the inner diameter of the vial and the thickness of the segments depends on the entered volume of the solution and the number of segments that the solution is divided into for simulation. Once the lists are established, the iterative core of the algorithm can start, and photoreaction progress is simulated.

The algorithm contains an additional option to be run with an arbitrary, fixed (wavelength- and concentration independent) apparent quantum yield. If this option is chosen, the apparent quantum yield value is requested from the user and then consequently used throughout the entire simulation. This allows the estimation of an apparent quantum yield from an experiment that was conducted with the 3D printed precision photoreactor. It is important to note that doing this can only lead to an estimate in case the quantum yield is wavelength- and/or concentration-dependent. If the option is not chosen, the algorithm will proceed as described above and as described in Figure S28.
As an example, the irradiation experiments that were carried out with LED 2 (λ_{max} = 343 nm), were used to determine the respective apparent quantum yield, refer to Section 2.6 of this Supplementary Information. In case of irradiation of 0.5 μmol A and 0.6 μmol NEM in 0.25 mL acetonitrile for 300 s with LED 2 in the 3D-printed precision photoreactor (5.3 mW), an arbitrary quantum yield of 0.03 leads to a predicted conversion of 20.2%. Comparison with the observed conversion of 18.6% (refer to Table S19) indicates that the arbitrarily set quantum yield can be refined, leading with a set value of

0.027 to a predicted conversion of 18.2%. The algorithm can therefore be used to estimate quantum yields based on LED experiments that were carried out with the 3D-printed precision photoreactor. The effect of the added competitive absorber HNBA can then be estimated using the apparent, fixed quantum yield of 0.027. In case of the irradiation of 0.5 μmol A and 0.6 μmol NEM and 0.38 μmol HNBA in 0.25 mL acetonitrile for 480 s with LED 2 in the 3D-printed precision photoreactor (5.3 mW), 14.8% conversion is predicted, while 17.3% is experimentally observed, refer to section 2.7 and Table S13. In contrast, the prediction using the wavelength- and concentration-dependent quantum yield map that was determined from the tunable laser experiments predicts 18.0% conversion. Thus, tunable laser experiments are best to characterize photochemical reactions, but good reactivity estimates can be made if a well-defined irradiation setup is used, such as the here introduced 3D-printed LED precision photoreactor.

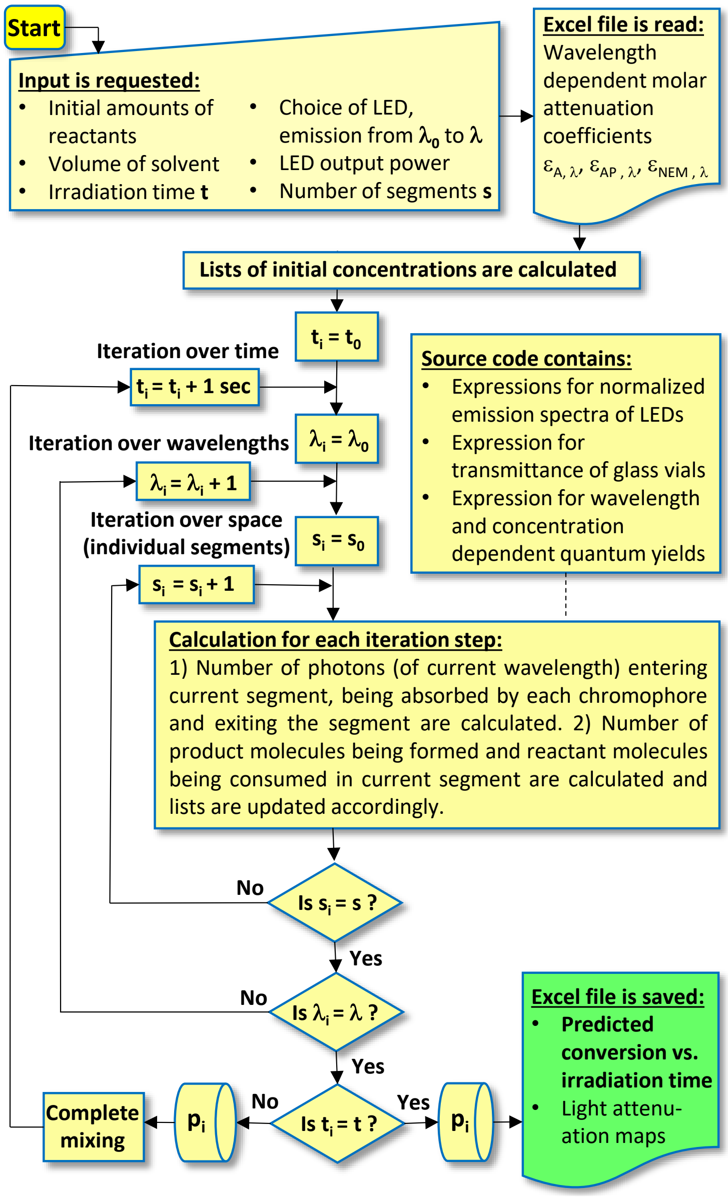

Figure S28: Flow chart for the predictive algorithm (LED induced conversion). Flow chart showing the algorithm that is used to quantitatively predict LED light induced photoreactivity. Manual input as well as extraction of data from an excel file allow the initial state to be defined, saved as lists containing amounts of each compound respectively corresponding to a stack of thin segments that the sample solution is divided into. The simulation then progresses with iterations over time, wavelengths and segments. Mathematical expressions hold the information shown in Figure 2, allowing the calculation of local changes to the compound distributions. After each simulated second, the overall conversion p_i is saved and a module simulating complete mixing of the solution is performed. Finally, the output data is saved, refer also to Figure 3 and Figure 4 in the main paper.

2.5 Simulation of wavelength-dependent selectivity of competing photoreactions

The above-described simulations are validated as a tool to predict the time-dependent conversion of an LED light induced experiment. The predicted result is found to be accurate, even if a further competitive absorber is added to the mixture before irradiation. The algorithm is written in a modular fashion, enabling modifications that allow more complex predictions. To evidence the potential that this platform technology has, it was attempted here to predict the wavelength-dependent selectivity of competing photoreactions. Such a prediction is key in the development of sequence-independent λ-orthogonal ligation systems when irreversible covalent links are formed for each reaction channel.

Based on the source code described in section 2.5 an algorithm was developed, which performs multiple simulations of photoreactions driven by monochromatic light with variation of the wavelength (tunable laser light) for each simulation that is run successively. Upon starting the algorithm, the wavelength range is requested, for which simulations are run in 0.5 nm steps. The algorithm further requests the input of the volume of the solution, amounts of each reactant and number of segments (analogously to the previous algorithm, the solution is divided into segments for simulation). Further a target conversion and a target photon count per laser pulse are requested. The algorithm specifically contains the wavelength-dependent quantum yields for both the reaction of A with NEM to AP as well as B with NEM to BP (refer to section 2.2 and previously published data[2]). The respective molar attenuation coefficients are read from an excel file before the actual simulation starts. For each wavelength, conversion over time is simulated for the two photoreactions taking place simultaneously (for wavelengths, where both substrates are known to be absorbing light and be reactive). Each simulation is stopped, when either of the two reaction channels reaches a defined target conversion value, e.g. 97%. Subsequently, the conversion is saved in a list for each of the two reactions as well as the number of simulated laser pulses to reach the target conversion. The resulting conversion can be plotted as conversion against the wavelength and constitutes a prediction of the selectivity, including the number of laser pulses that are required to reach this outcome. A flowchart describing the algorithm is shown in Figure S29.

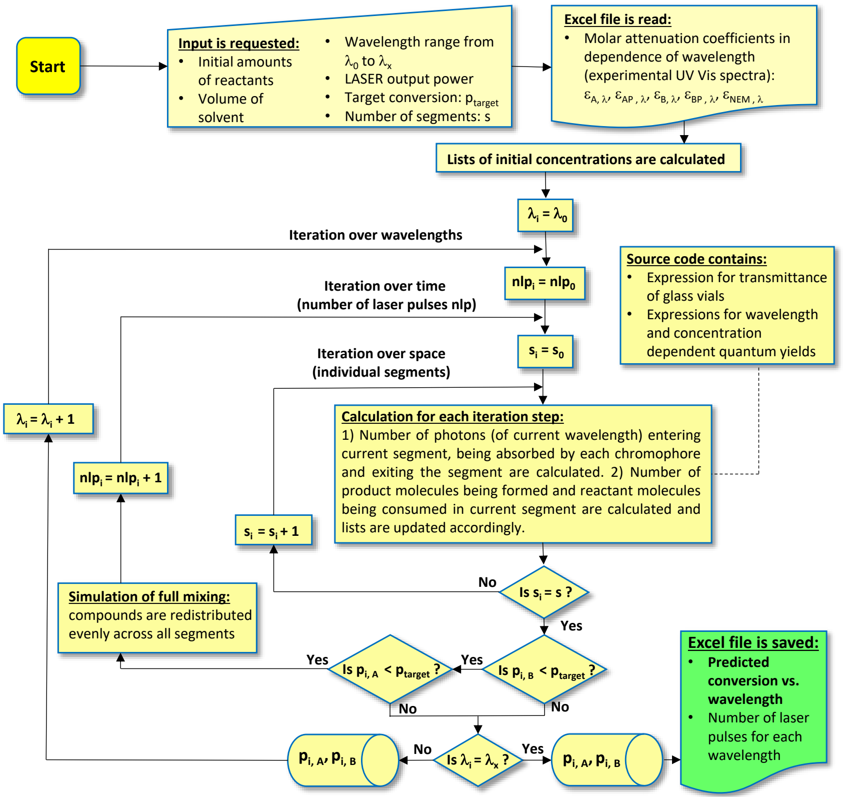

Figure S29: Flow chart for the predictive algorithm (Laser induced conversion and selectivity). Flow chart showing the algorithm that is used to quantitatively predict the wavelength dependent selectivity of competing photoreactions. Manual input as well as extraction of data from an excel file allow the initial state to be defined, saved as lists containing amounts of each compound respectively corresponding to a stack of thin segments that the sample solution is divided into. The simulation then progresses for each wavelength with iterations over the number of simulated laser pulses and segments. Mathematical expressions hold the information shown in Figure 2, allowing the calculation of local changes to the compound distributions. After each simulated laser pulse, the overall conversion $\mathbf{p_{i,\,A}}$ is $\mathbf{p_{i,\,B}}$ is saved and a module simulating complete mixing of the solution is performed. Finally, the output data is saved, refer also to Figure 5 in the main paper.

2.6 Conversion of photoligation of A with NEM using LED 2 and LED 3

A stock solution containing A (2.0 mmol L^{-1}), NEM (2.4 mmol L^{-1}) and TMB (2.0 mmol L^{-1}) in CD_3CN is prepared. Each 0.25 mL are transferred to a glass vial containing a stir bar. The vial is crimped, and the sample solution is deoxygenated by a stream of nitrogen for 10 minutes. Each sample is irradiated either with LED 2 or 3 for the duration as indicated in Table S19 using the 3D printed batch photoreactor while being stirred at 500 rpm. The irradiation procedure starts with the manual shutter being closed before the sample is inserted, refer also to Figure S1. Before the irradiation starts, the LED is turned on for 2 min, while the solution is already being stirred at 500 rpm and the fan is turned on. Subsequently, irradiation is started by pulling the manual shutter out, allowing the sample vial to drop by 1 mm into its position for irradiation. After the designated irradiation time, the manual shutter is pushed in (lifting the sample up by 1 mm) and thereby ensuring that no further light reaches the sample. Finally, the LED, fan and magnetic stirrer are turned off. Conversion is determined by ^{1}H-NMR spectroscopy, refer also to Figure S30. Conversion is both determined from the relative integrals of relevant resonances of A and AP ($p_{A\rightarrow AP}$) and the relative integrals of A and the internal standard TMB ($p_{A\rightarrow \Sigma AP}$, utilizing the spectrum of the stock solution). The deviation between those conversion values, respectively, is within the accuracy of the NMR measurement. This finding and the fact that no other resonances are found in the spectrum of irradiated samples, indicate that the photoreaction proceeds with a single reaction trajectory under these conditions.

Table S19: Irradiation times with LED 2 and 3 as well as predicted conversion and measured conversion by ^{1}H-NMR spectroscopy.

entry	LED	λ_{max} of LED	Irradiation time / s	Predicted conversion / %	$p_{A\rightarrow AP}$ (NMR) / %	$p_{A\rightarrow \Sigma AP}$ (NMR) / %
1	2	343	300	21.0 (17.8 < p < 24.6)	18.2	19.0
2	2	343	300	21.0 (17.8 < p < 24.6)	18.7	20.5
3	2	343	300	21.0 (17.8 < p < 24.6)	18.8	22.0
4	2	343	600	45.1 (37.7 < p < 53.3)	44.4	47.5
5	2	343	600	45.1 (37.7 < p < 53.3)	42.7	45.0
6	2	343	600	45.1 (37.7 < p < 53.3)	48.3	50.0
7	2	343	900	71.8 (60.0 < p < 83.8)	76.8	77.0
8	2	343	900	71.8 (60.0 < p < 83.8)	70.3	71.0
9	2	343	900	71.8 (60.0 < p < 83.8)	72.1	72.0
10	3	380	45	23.0 (19.6 < p < 26.8)	28.4	29.5
11	3	380	45	23.0 (19.6 < p < 26.8)	28.3	30.0
12	3	380	45	23.0 (19.6 < p < 26.8)	28.4	31.0
13	3	380	90	48.1 (40.8 < p < 56.0)	54.2	56.0
14	3	380	90	48.1 (40.8 < p < 56.0)	53.0	52.5
15	3	380	90	48.1 (40.8 < p < 56.0)	58.5	60.0
16	3	380	135	73.0 (62.7 < p < 82.6)	77.2	77.5
17	3	380	135	73.0 (62.7 < p < 82.6)	80.2	80.5
18	3	380	135	73.0 (62.7 < p < 82.6)	85.3	85.5

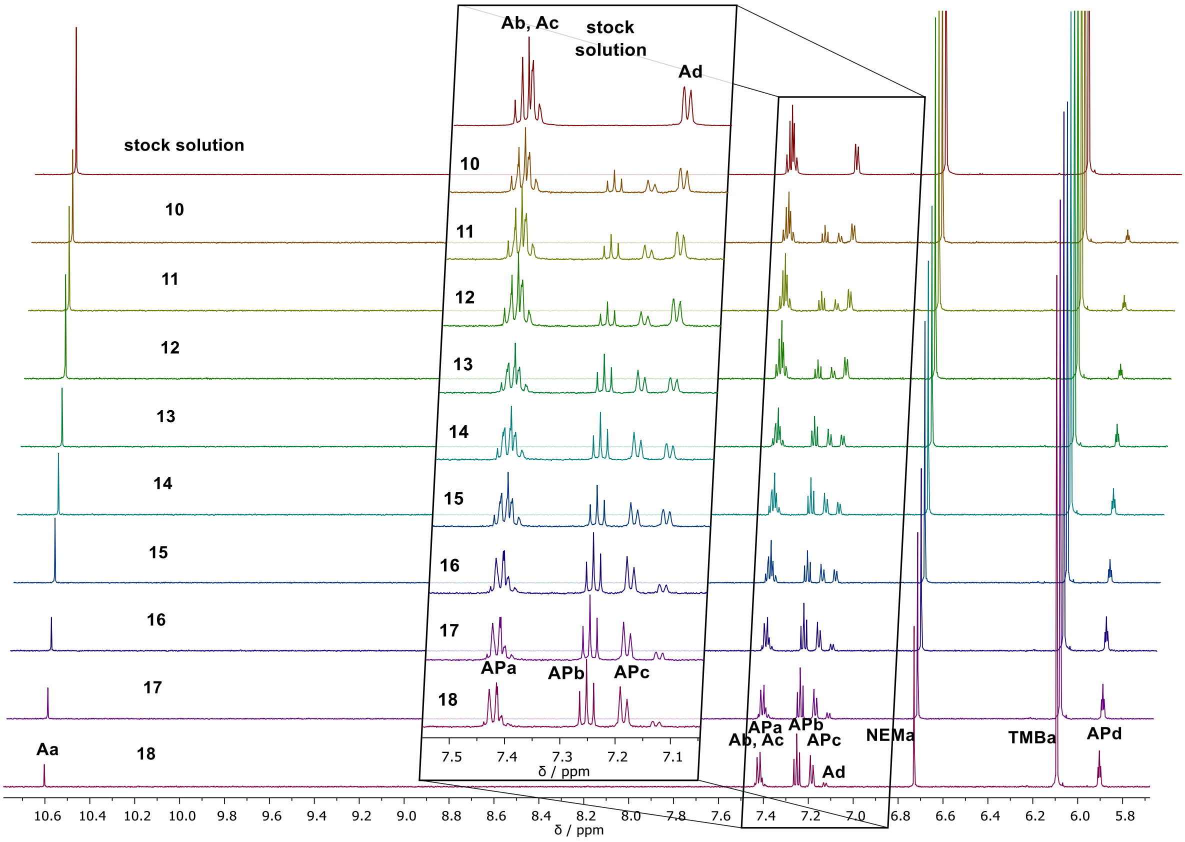

Figure S30: ^{1}H-NMR spectra of samples irradiated with LED 3 with irradiation times shown in Table S12.

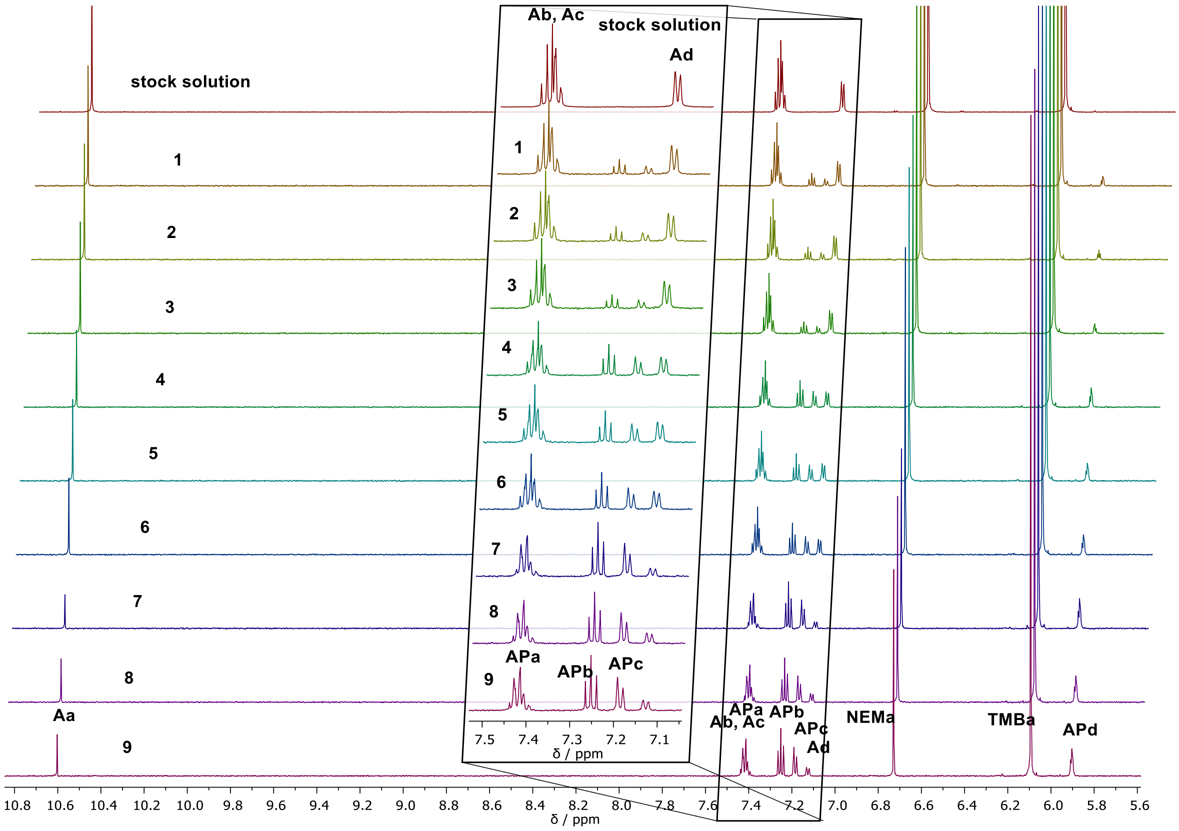

Figure S31: ^{1}H-NMR spectra. ^{1}H-NMR spectra of the samples that were irradiated with 300, 600 and 900 seconds with LED 2, respectively, as well as the stock solution that these samples were prepared from, refer also to Table S12.

2.7 Conversion of photoligation of A with NEM in the presence of HNBA using LED 2

A stock solution containing A (2.0 mmol L^{-1}, 1.0 eq.), NEM (2.4 mmol L^{-1}, 1.2 eq.), HNBA (1.3 mmol L^{-1}, 0.65 eq.) and TMB (2.0 mmol L^{-1}, 1.0 eq.) in CD_3CN is prepared. Each 0.25 mL are transferred to a glass vial containing a stir bar. The vial is crimped, and the sample solution is deoxygenated by a stream of nitrogen for 10 minutes. Each sample is irradiated with LED 2 employing the 3D printed batch photoreactor for the irradiation times as shown in Table S20.

Table S20: Irradiation times for the experiments with addition of HNBA and irradiation with LED 2 as well as predicted conversion and measured conversion by ^{1}H-NMR spectroscopy.

entry	LED	λ_{max} of LED	Irradiation time / s	Predicted conversion / %	$p_{A\rightarrow AP}$ (NMR) / %	$p_{A\rightarrow \Sigma AP}$ (NMR) / %
1	2	343	480	18.0 (15.4 < p < 20.9)	17.8	23.0
2	2	343	480	18.0 (15.4 < p < 20.9)	15.6	17.0
3	2	343	480	18.0 (15.4 < p < 20.9)	18.5	22.0
4	2	343	1200	45.2 (38.7 < p < 52.0)	43.3	49.0
5	2	343	1200	45.2 (38.7 < p < 52.0)	43.5	49.0
6	2	343	1200	45.2 (38.7 < p < 52.0)	41.6	43.0
7	2	343	2100	75.0 (65.9 < p < 83.2)	75.6	78.0
8	2	343	2100	75.0 (65.9 < p < 83.2)	74.0	75.0
9	2	343	2100	75.0 (65.9 < p < 83.2)	79.0	82.0

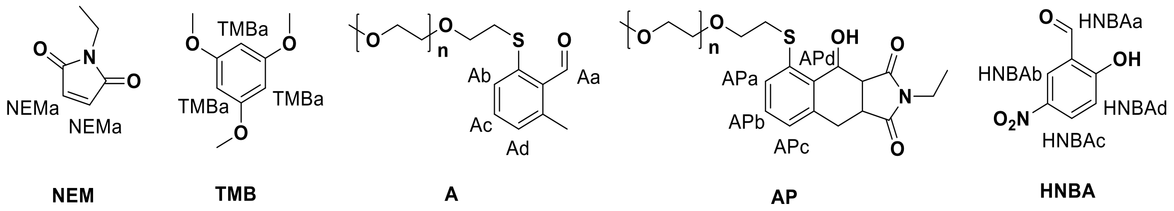

Figure S32: Structures and assignment as reported in the NMR spectra shown in Figure S33.

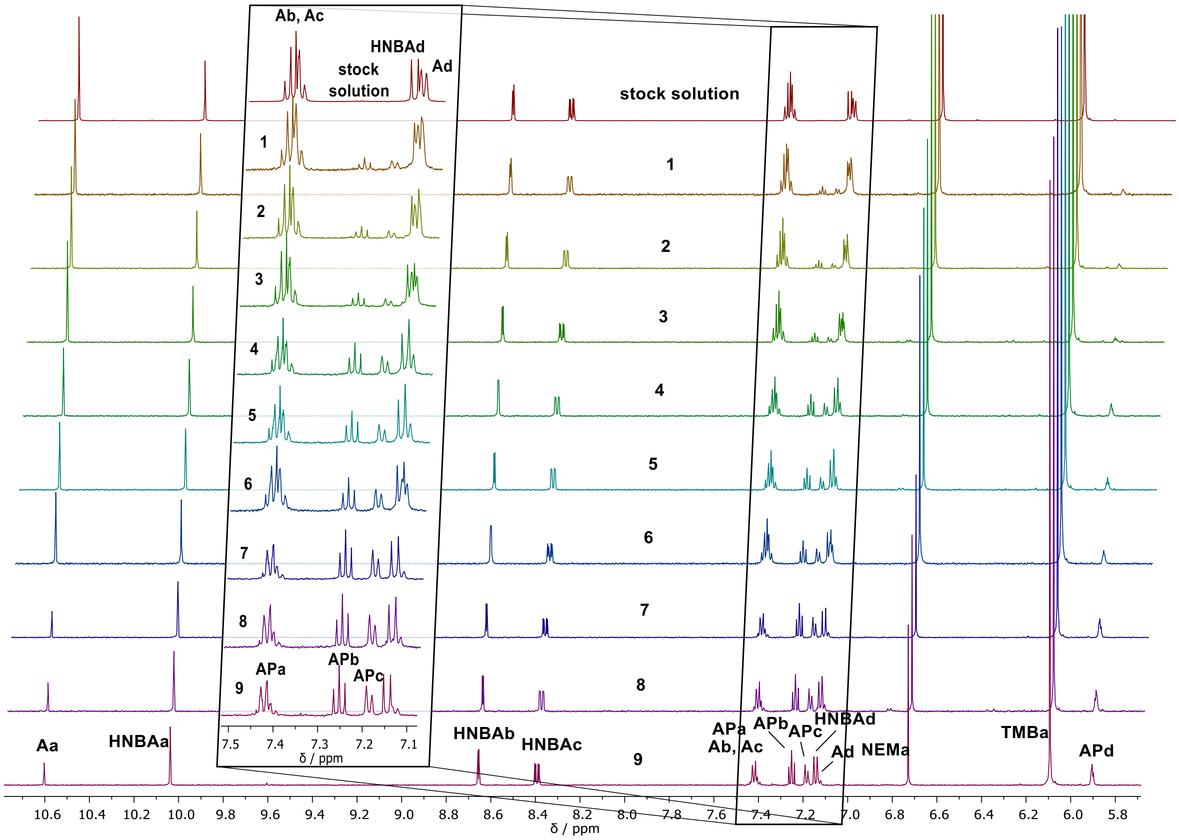

Figure S33: ^{1}H-NMR spectra of the samples containing HNBA. Samples were irradiated with 480, 1200 and 2100 seconds with LED 2, respectively. Shown as well is the stock solution that these samples were prepared from, refer also to Table S20.

2.8 Additional predictions and experiments with LED 1 and LED 4

For additional experiments as discussed here, stock solutions equal to the ones used in previous experiments (refer to section 2.6 of this Supplementary Information) are used and treated analogously. Irradiation of **A** and **NEM** with LED 1 (λ_{max} = 285 nm) revealed that with increasing conversion, side reactions became more and more significant. Thus, this LED is not equally included in the above study for the validation of the predictive framework. Table S21 shows that with increasing conversion (285 nm irradiation) a significant discrepancy between $p_{A\rightarrow AP}$ and $p_{A\rightarrow \Sigma AP}$ is observed, indicating that consumption of **A** is not exclusively leading to the formation of **AP**, refer also to Figure S35. Side products were not investigated further, because they are only formed during irradiation with LED 1 and for most applications of this photoligation longer wavelengths are employed. Despite the side reactions, the observed conversion does not deviate much from predicted values, whereas prediction and experiment match increasingly less for longer irradiation times.
In case of the use of LED 4, a very slow reaction was predicted, preventing that high conversion values can be reached on reasonable timescales. Thus, only three experiments with an irradiation time of each 20h was conducted. The very small overlap between the emission spectrum of LED 4 and the absorption spectrum of A as well as the very low molar attenuation coefficient as well as quantum yield at the respective wavelength cause a very slow reaction progress. Yet, through conducting the computational prediction, an experiment was designed (overnight irradiation), which shows that this LED can lead to measurable conversion.
The very small absorbance of **A** at 420 nm as well as the very small overlap between emission spectrum of LED 4 and the absorbance of **A** make it challenging to predict the outcome of the reaction. The respective simulated light attenuation maps, refer to Figure S34, emphasize this further. Barely any change in the light attenuation map is observed, yet the computational simulation correctly predicts conversion for the experiment that was conducted. It should be noted here, that a small error in the acquisition of the emission spectrum of the LED could potentially lead to a significant error in the prediction, as the method relies on correctly calculating the fraction of emitted photons in the wavelength range of 400 nm to 420 nm, despite the emission spectrum being centered around 440 nm. The emission spectrum of LED 4 was obtained seven times and averaged over those measurements to reduce the chance of introducing a significant error.

Table S21: Irradiation times with LED 1 and 4 as well as predicted conversion and measured conversion by ^{1}H-NMR spectroscopy.

entry	LED	λ_{max} of LED	Irradiation time / s	Predicted conversion / %	$p_{A\rightarrow AP}$ (NMR) / %	$p_{A\rightarrow \Sigma AP}$ (NMR) / %
1	1	285	4500	30.7 (21.2 < p < 42.3)	26.8	34.5
2	1	285	4500	30.7 (21.2 < p < 42.3)	27.2	35
3	1	285	4500	30.7 (21.2 < p < 42.3)	21.7	28
4	1	285	9000	62.3 (43.3 < p < 81.9)	41.1	57
5	1	285	9000	62.3 (43.3 < p < 81.9)	41.6	55.5
6	1	285	9000	62.3 (43.3 < p < 81.9)	50.3	68
7	1	285	13500	87.6 (65.1 < p < 98.2)	50.9	68.5
8	1	285	13500	87.6 (65.1 < p < 98.2)	52.2	71
9	1	285	13500	87.6 (65.1 < p < 98.2)	65.6	82
10	4	440	72000	16.6 (13.7 < p < 19.8)	14.8	17.5
11	4	440	72000	16.6 (13.7 < p < 19.8)	19.8	24.5
12	4	440	72000	16.6 (13.7 < p < 19.8)	17.6	22

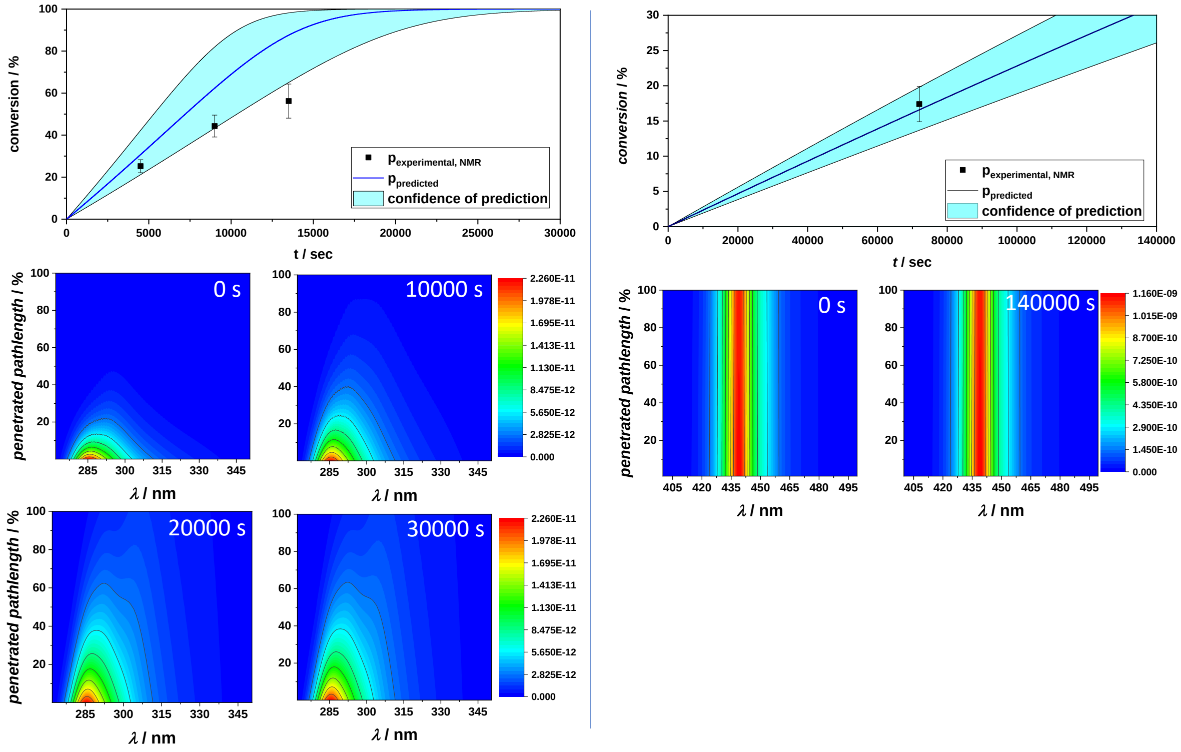

Figure S34: Predicted and observed conversion for experiments with LED 1 (left) and LED 4 (right). The light attenuation maps are simulated respective to the predicted conversion trace. In case of LED 4, only 140000 seconds of irradiation time were simulated, thus only the initial light attenuation map and one after 140000 seconds is displayed here. The experimentally observed conversion is the average of three replicates with the error bar indicating the found standard deviation.

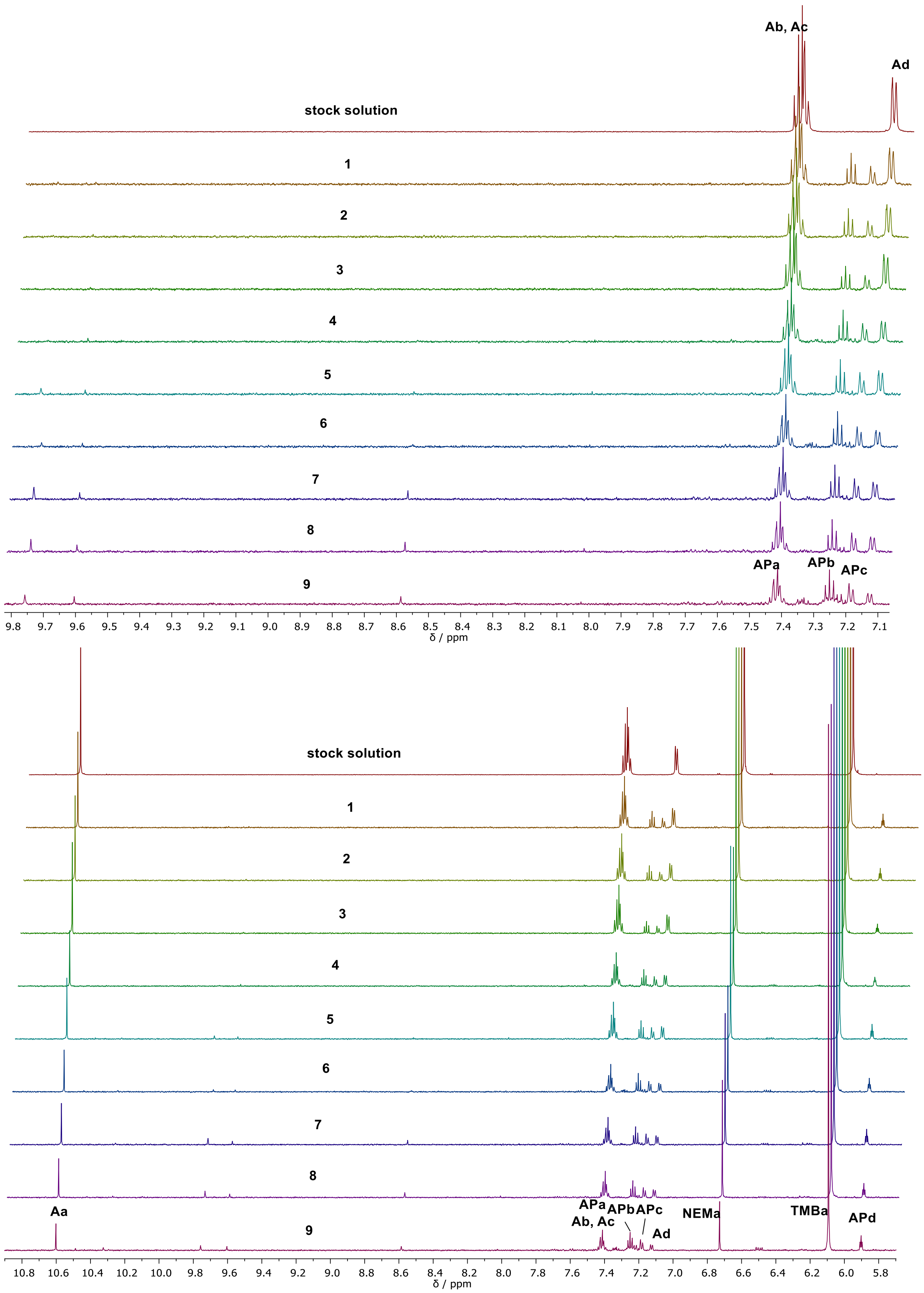

Figure S35: ^{1}H-NMR spectra of samples irradiated with LED 1 and the stock solution, respectively. Above: Expansion of the spectrum to visualise relevant areas, where changes occur. Below: Same spectrum with wider expansion to show relevant signals.

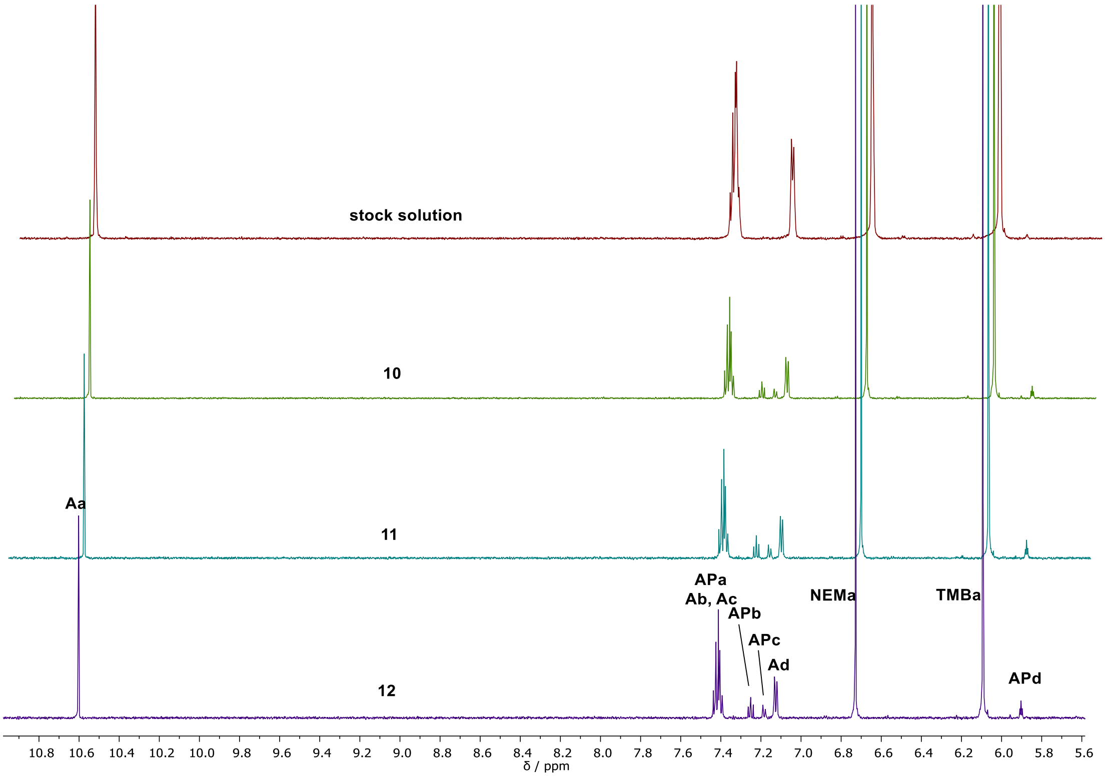

Figure S 36: ^{1}H-NMR spectra of the stock solution used here and the respective samples after irradiation with LED 4 for 20 h.

2.9.1 Sequence-independent λ-orthogonal photoligation employing two photoenol ligation reactions

According to predictions outlined in Figure 5 and section 2.4 of this Supplementary Information, the conversion of **A** and **B** with **NEM** can be carried out with the highest expected selectivity within the range of 300 nm to 330 nm. A higher conversion is expected to benefit the selective conversion of **B** in presence of **A**, due to the concentration dependence of the quantum yields of **A**.

A stock solution containing 2.8 μmol (1.0 eq., 6.4 mmol L^{-1}) **A**, 2.8 μmol (1.0 eq., 6.4 mmol L^{-1}) **B**, 6.8 μmol (2.4 eq., 15 mmol L^{-1}) **NEM** and 1.1 μmol (0.4 eq., 2.5 mmol L^{-1}) Trimethoxybenzene **TMB** in 0.44 mL CD_3CN was prepared. The stock solution was characterized *via* ^{1}H-NMR spectroscopy, refer to Figure 6 and Figure S38.
0.11 mL (containing 0.7 μmol of each **A** and **B**, 1.7 μmol **NEM** and 0.27 μmol **TMB**) of the stock solution was transferred to a glass vial and deoxygenated by passing a stream of nitrogen for 10 min. The sample was irradiated with a tunable laser at 325 nm (184 μJ per pulse) for 4 min and 24 s (264 seconds irradiation time, 5280 laser pulses, each 0.5 nmol photons reaching the sample glass vial per pulse). Conversion of **A** and **B** was determined by ^{1}H-NMR spectroscopy, refer to Figure 6 and Figure S39.
0.11 mL of the same stock solution was diluted with CD_3CN to a total volume of 0.5 mL. The sample solution was transferred to a glass vial and deoxygenated by passing a stream of nitrogen for 10 min. The sample was irradiated with a tunable laser at 415 nm (1.0 mJ per pulse) for 100 min (6000 seconds irradiation time, 120000 laser pulses, each 3.5 nmol photons reaching the sample glass vial per pulse). Conversion of **A** and **B** was determined by ^{1}H-NMR spectroscopy, refer to Figure 6 and Figure S40.

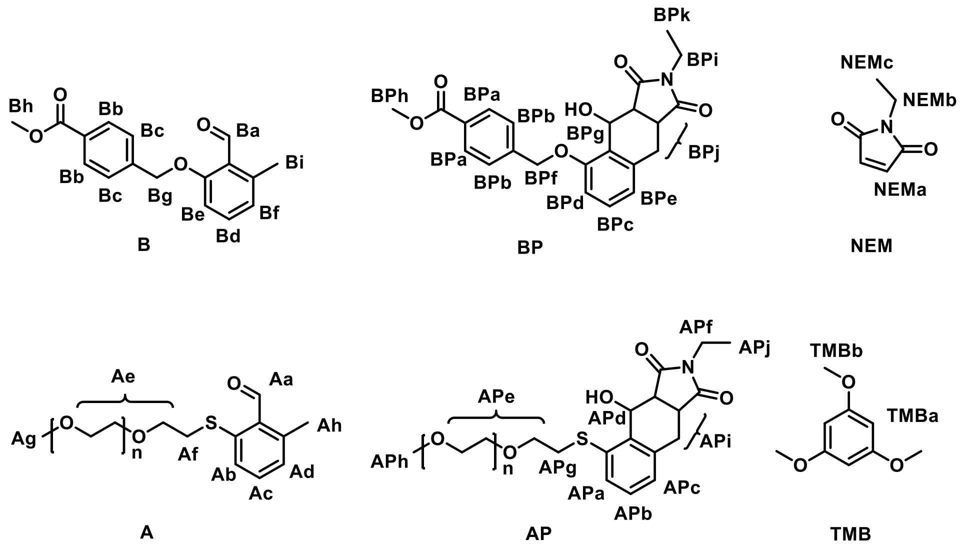

Figure S37: Structures and assignment of resonances in NMR spectra shown in Figure S31, Figure S32 and Figure S33.

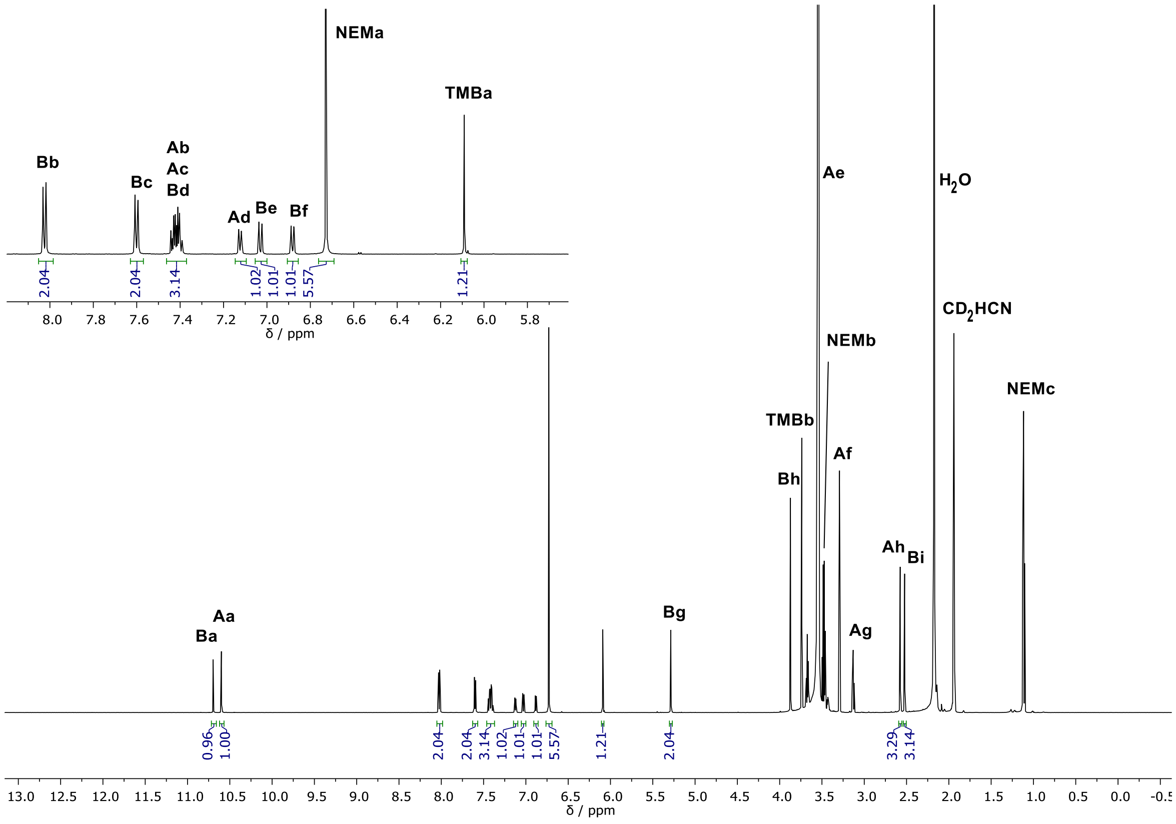

Figure S38: ^{1}H-NMR spectrum of the stock solution containing A, B, NEM and TMB.

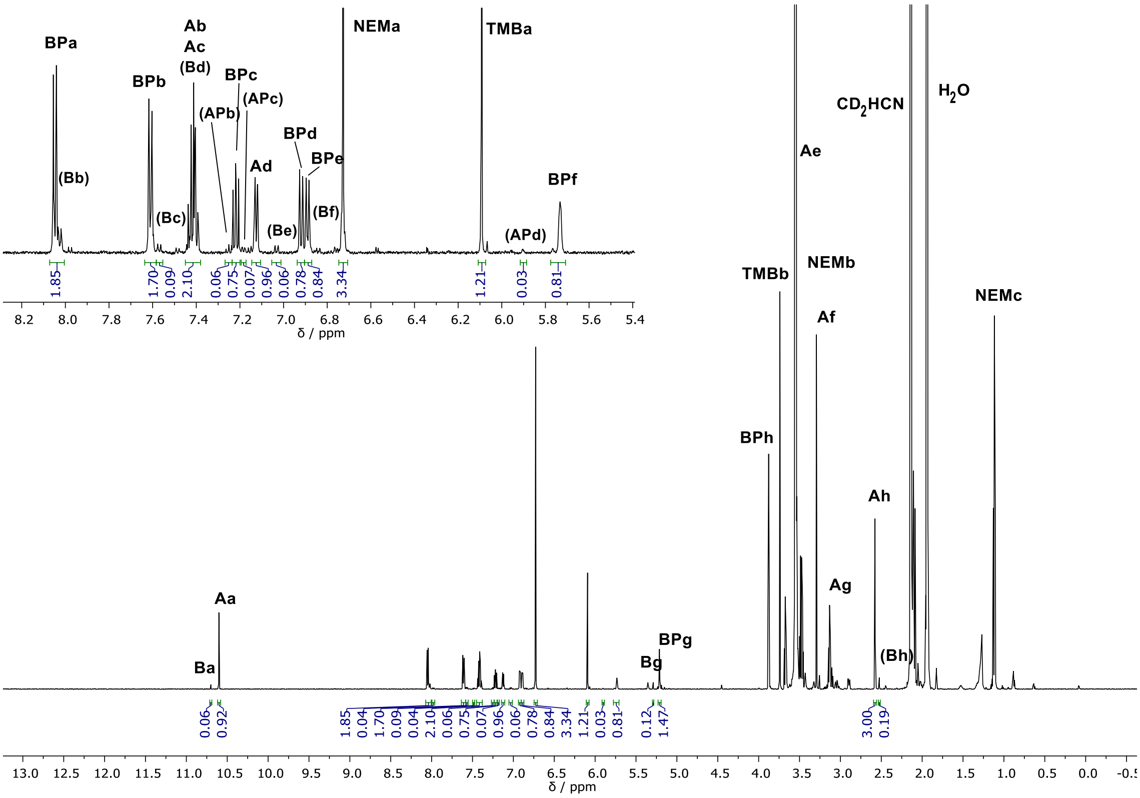

Figure S39: ^{1}H-NMR spectrum of the sample after irradiation with 325 nm.

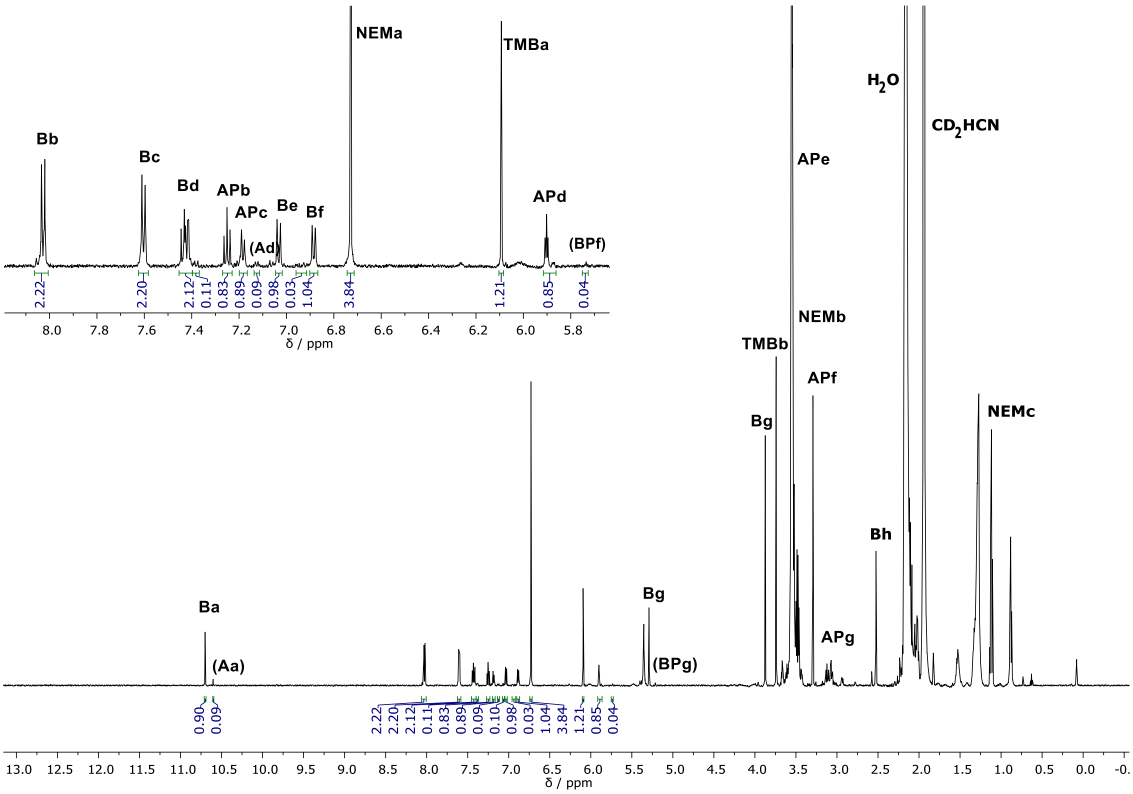

Figure S40: ^{1}H-NMR spectrum of the sample after irradiation with 415 nm.

2.9.2. Quantum yields of 2-(dodecylthio)-6-methylbenzaldehyde C and unprecedented selectivity between C and tetrazole D

The quantum yields of the reaction of C with *N*-ethylmaleimide to the cycloadduct CP were investigated with the tunable laser in analogy to the experiments described in section 2.2.1, whereas here the quantum yields were only investigated at wavelengths 285 nm and 360 nm.
0.96 mg (3.0 μmol, 1 eq.) 2-(dodecylthio)-6-methylbenzaldehyde C, 0.43 mg (3.45 μmol, 1.15 eq.) *N*-ethylmaleimide NEM and 0.50 mg (3.0 μmol, 1.0 eq.) 1,3,5-trimethoxybenzene TMB were dissolved in 1.5 mL CD_3CN. Each 0.25 mL were transferred to a glass vial and deoxygenated with a stream of nitrogen for 10 min, followed by irradiation with the laser according to the parameters listed in Table S22. Conversion was determined by ^{1}H-NMR spectroscopy.

Table S22: Parameters for tunable laser experiments for the determination of quantum yields of **C** at 285 nm and 360 nm.

λ / nm	V_{CD3CN} / mL	$t_{irradiation}$ / s	E_{pulse} / μJ	$n_{p, total}$ / μmol	n_{TMB} / μmol	n_A / μmol	n_{AP} / μmol	n_{NEM} / μmol	$p_{A→AP}$ / %	$p_{A→ΣAPx}$ / %	$Φ_{A→AP}$	$Φ_{A→ΣAPx}$
285	0.25	1500	255	5.09	0.5	0.39	0.055	0.24	12.5	23.0	0.0103	0.022
285	0.25	1500	230	4.60	0.5	0.38	0.053	0.23	12.5	25.0	0.0115	0.026
285	0.25	1500	205	4.10	0.5	0.37	0.057	0.24	13.2	25.5	0.0136	0.031
360	0.25	180	206	1.85	0.5	0.48	0.027	0.45	5.2	3.5	0.0077	0.005
360	0.25	180	205	1.84	0.5	0.47	0.025	0.44	5.1	6.5	0.0074	0.0093
360	0.25	180	204	1.84	0.5	0.47	0.030	0.44	6.0	6.5	0.0087	0.0094

0.32 mg (0.99 μmol, 1 eq.) 2-(dodecylthio)-6-methylbenzaldehyde C, 2.18 mg (0.99 μmol, 1 eq.) α-methyl-ω-4-(2-(4-methoxyphenyl)-2H-tetrazol-5-yl)benzamido poly(ethylene glycol) D, 0.43 mg (3.45 μmol, 1.15 eq.) *N*-ethylmaleimide NEM and 0.50 mg (3.0 μmol, 1.0 eq.) 1,3,5-trimethoxybenzene TMB were dissolved in 1.5 mL CD_3CN. Each 0.25 mL were transferred to a glass vial and deoxygenated with a stream of nitrogen for 10 min, followed by irradiation with LED 1 in the 3D-printed precision photoreactor for 30 (60, 240) minutes (The LED output was controlled with a regulated power supply – 6.8 V, 0.03 A; LED power: 1.0 mW). Conversion was determined by ^{1}H-NMR spectroscopy, refer to Figure S41 and Figure S42.

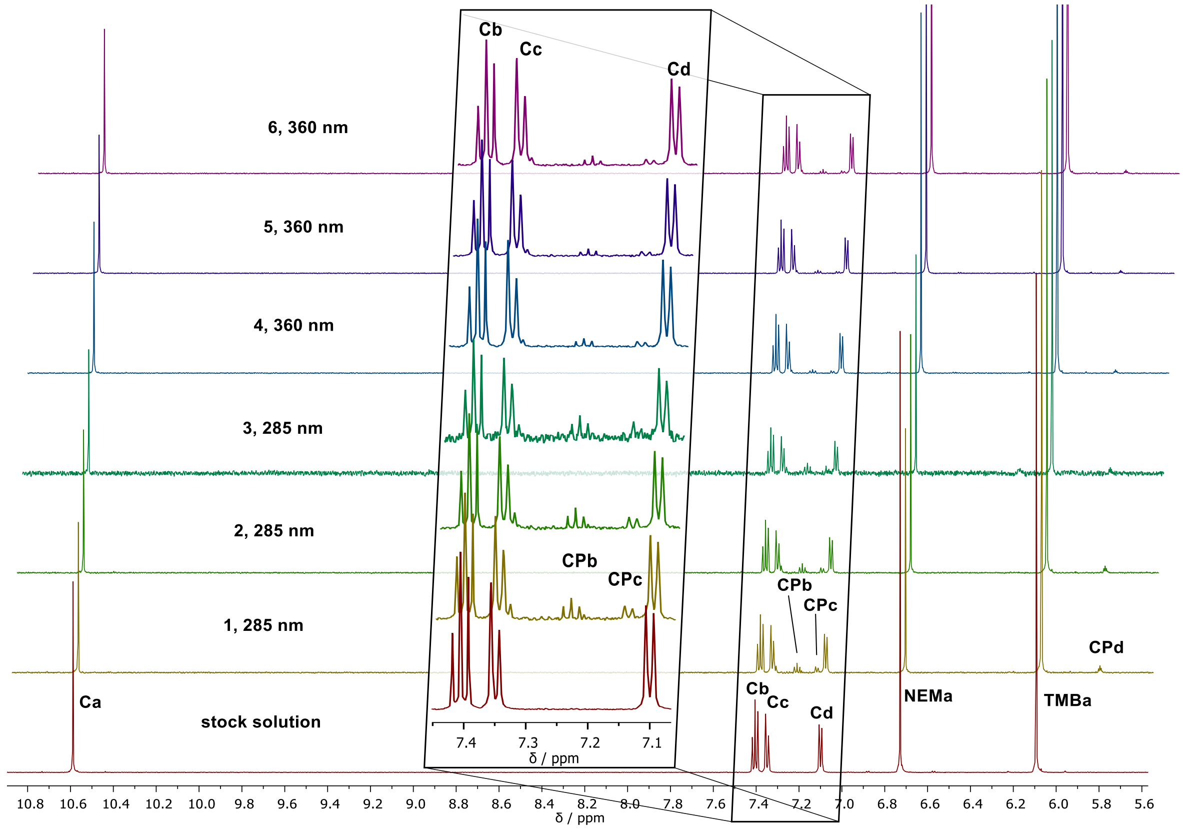

Figure S41: ^{1}H-NMR spectra of samples irradiated with a defined photon count at 285 nm and 360 nm. The structures and assignments of signals are shown in Figure S42.

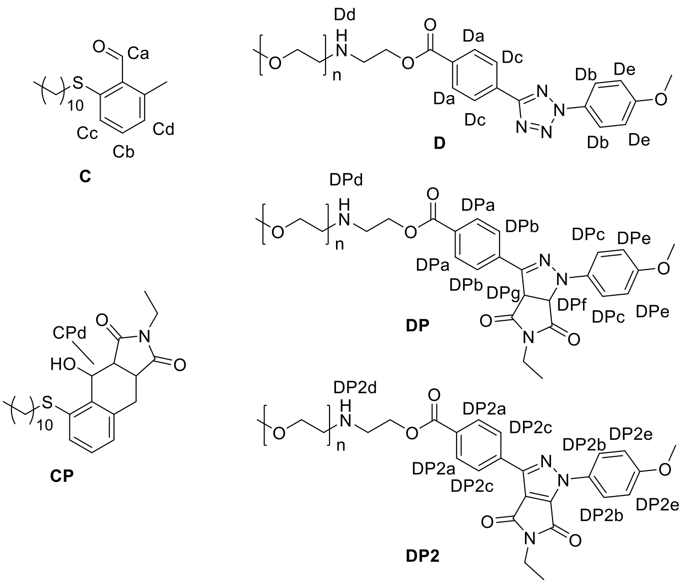

Figure S42: Structures and ^{1}H-NMR assignments for Figure S41 and Figure S43.

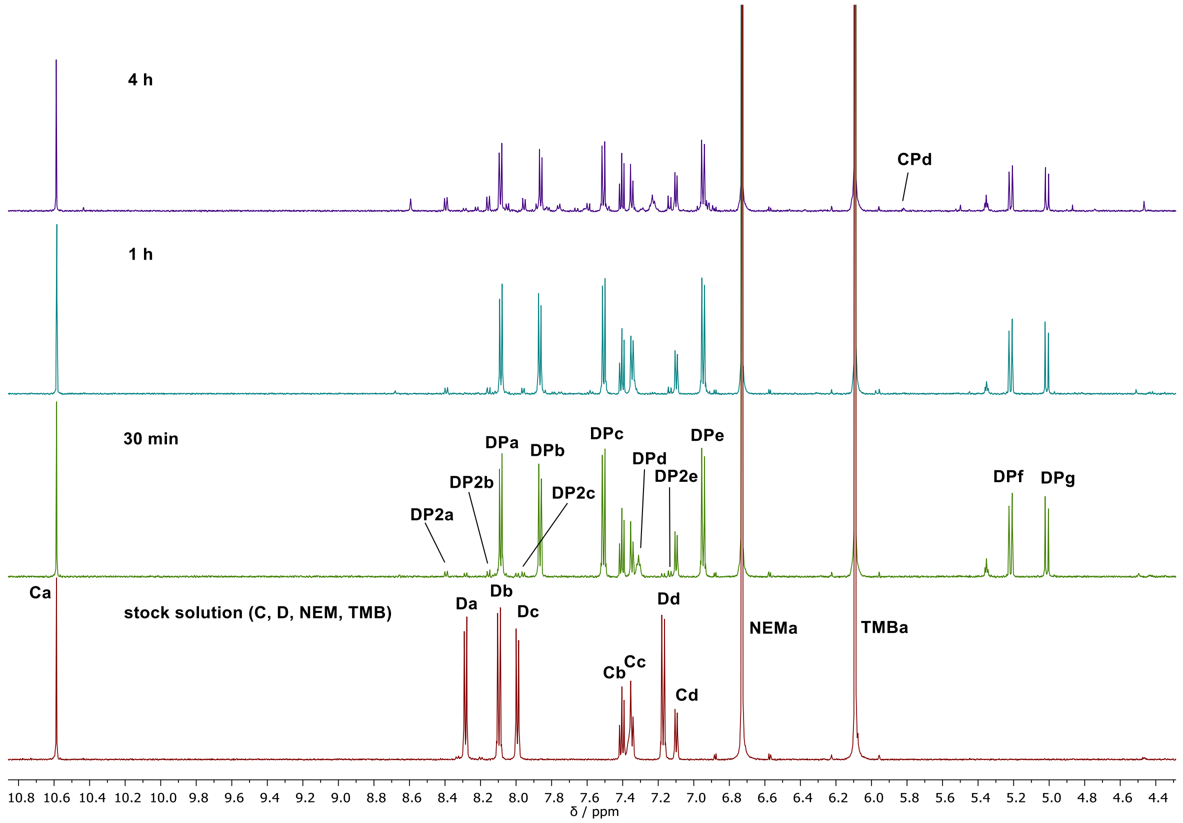

Figure S43: ^{1}H-NMR spectra of a solution of C, D, NEM and TMB, before (bottom spectrum) and after 30 minutes, 1 hour and 4 hours of irradiation with LED 1 (λ_{max} = 285 nm). The respective structures and assignments of signals are shown in Figure S42.

2.9.3. Prediction and experimental observation of selectivity between tetrazole D and *o*-methylbenzaldehyde B

The following simulations and experiments were carried out to investigate, whether the predictive algorithms are also capable of making valid predictions in case of a combination of substrates that can be expected to lead to a less selective outcome than observed in previous experiments shown in sections 2.9.1 and 2.9.2. Irradiation of a mixture of tetrazole D and *o*-methylbenzaldehyde B with wavelengths at which both compounds significantly absorb light generally leads to an outcome with less selectivity than the above-mentioned experiments, as both compounds exhibit a comparably high reaction quantum yield in this wavelength range. For comparability, the (monochromatic) wavelength of irradiation is chosen as 313 nm, as here the molar attenuation coefficient of D is 3.3 times higher than B. This is in analogy to the ratio of the molar attenuation coefficient of B vs. A at 325 nm of 3.3. Thus, any difference in predicted and observed selectivity between these experiments can be attributed more to the change in reaction quantum yield, rather than the ratio of the molar attenuation coefficients of the starting materials. For the prediction the reaction quantum yield of tetrazole D is estimated to be equal or greater than 0.55, as reported previously.[1] This value may slightly underestimate the reactivity of D.

A simulation with the following input parameters was run to predict conversion of each reaction channel: n_B = 0.7 mmol; n_D = 0.75 mmol; n_{NEM} = 1.7 mmol; wavelength range: 285 nm – 340 nm; target conversion: 25%; Φ_D = 0.55. The glass transmittance, absorbance and the reaction quantum yield of B is used as reported for the other predictions. The confidence of the prediction is estimated through simulation with varied input parameters (reaction quantum yield: ±10%, transmittance: ±3%). This estimation does not take into account that the reaction quantum yield of tetrazole D could in fact be higher than 0.61. Due to the strong absorbance of the pyrazoline cycloadduct DP, the reaction quantum yield may be underestimated, if a value of 0.55 ± 10% is used.[1,2] The predicted conversion for each reaction and the experimentally observed conversion values are shown in Figure S44. In each case, the simulation proceeds until either reaction reaches 20% conversion. Thus, for varied input parameters (reaction quantum yields and transmittance), the respective number of required laser pulses and conversion of B to BP varies.

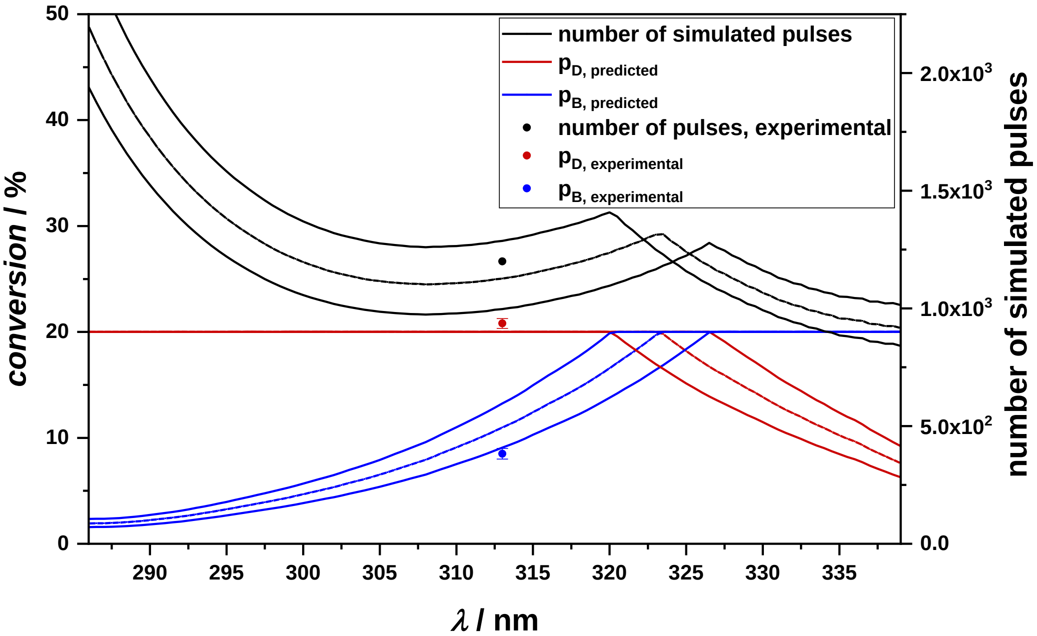

Figure S44: Predicted and observed selectivity. Red line: conversion of **D** to **DP**; blue line: conversion of **B** to **BP**; black line: number of predicted laser pulses to reach the target conversion of 20%. The values for the experimental conversion p_B and p_D are averages of three experiments and the error bar shows the standard deviation of these values. The confidence of the prediction is indicated by the three lines for each predicted, wavelength-dependent conversion values. These are the result of three simulations that were conducted with varied input parameters to take into account the average error of the quantum yield values (15%) and the average error of glass transmittance (3%).

4.95 mg (2.25 µmol, 1.08 eq.) D, 0.59 mg (2.1 µmol, 1.0 eq.) B, 0.62 mg (5.0 µmol, 2.39 eq.) *N*-ethylmaleimide NEM and 0.34 mg (2.05 µmol, 0.98 eq.) 1,3,5-trimethoxybenzene TMB were dissolved in 0.9 mL CD_3CN. The stock solution was characterized via ^{1}H-NMR spectroscopy, refer to Figure S44. Each 0.3 mL of this solution, containing 0.7 µmol B, 0.75 µmol D, 1.7 µmol NEM and 0.68 µmol TMB were transferred to a crimped glass vial containing a stir bar and the solution was deoxygenated by a stream of nitrogen gas for 10 minutes. The samples were irradiated with a tunable laser for 60

seconds at 313 nm at a target laser energy of 194 mJ/pulse (1200 pulses, total number of photons transmitted through the glass bottom into the sample solution: n_p = 0.43 mmol) while being stirred at 500 rpm. Conversion was determined via ^{1}H-NMR spectroscopy, refer to Figure S45.

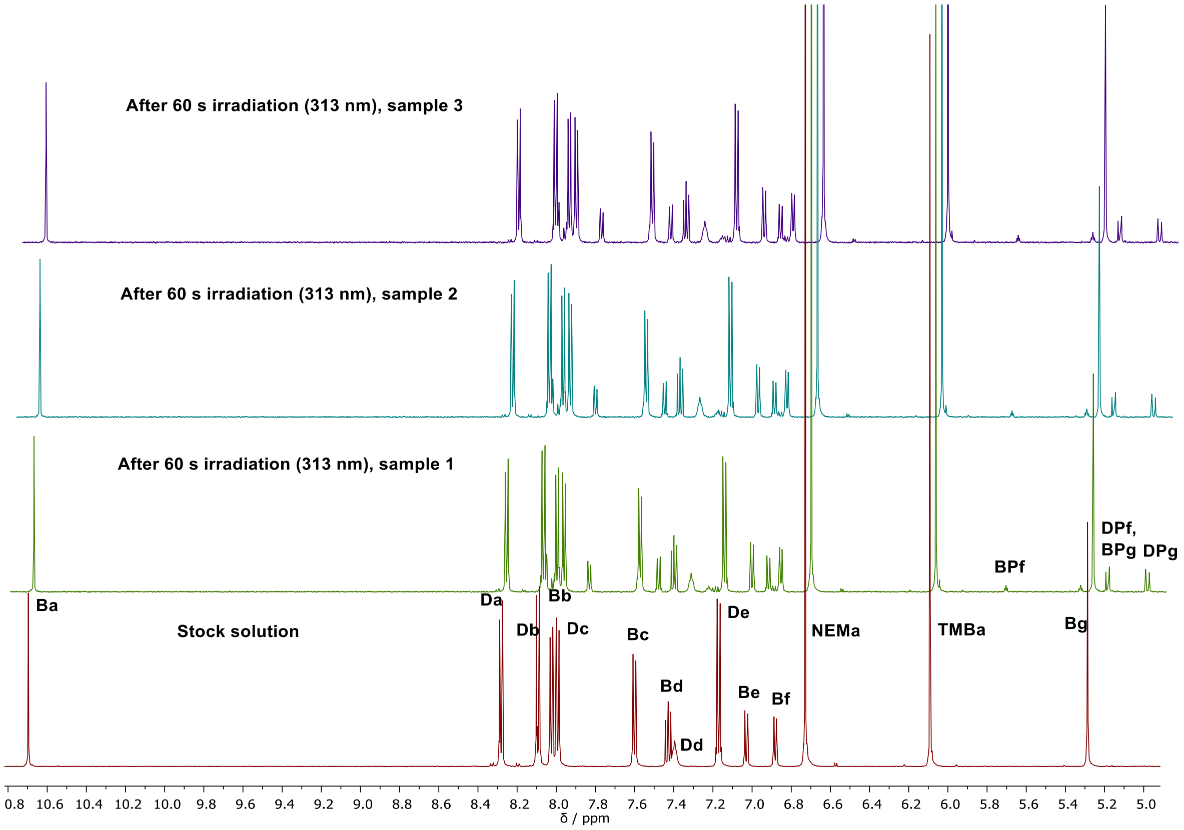

Figure S45: ^{1}H-NMR spectra of the stock solution and samples after irradiation with 313 nm for 60 s. An expansion of the same spectra from 8.35 to 6.85 ppm is shown in Figure S46.

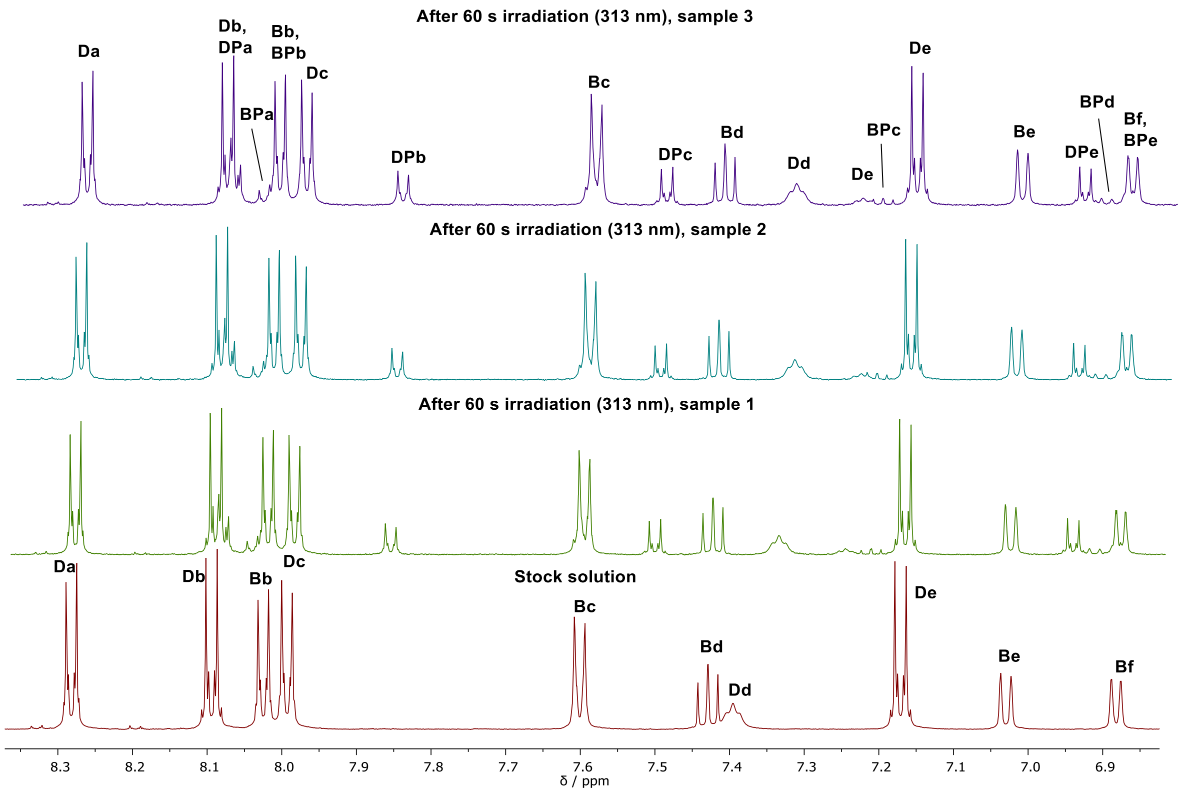

Figure S46: Expansion of ^{1}H-NMR spectra shown in Figure S45.

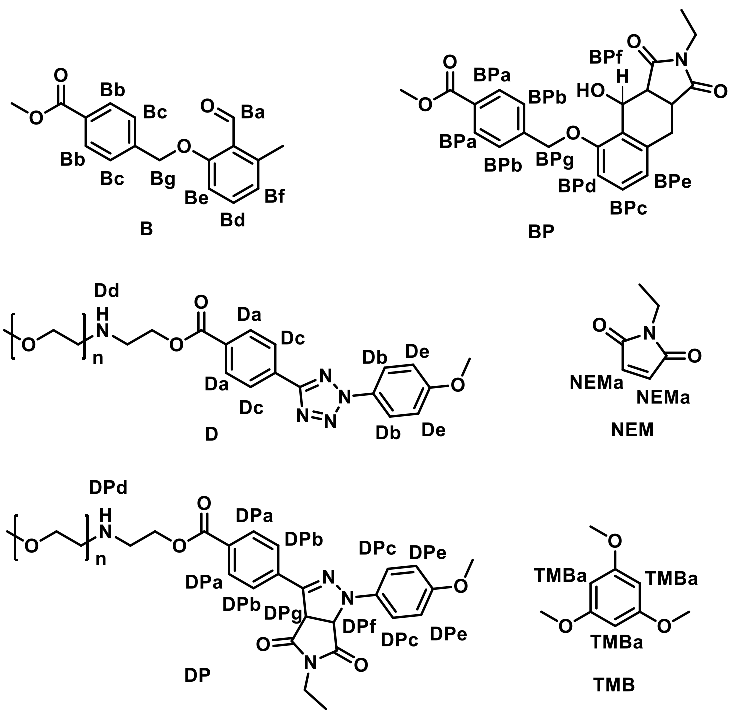

Figure S47: Structures and ^{1}H-NMR assignments for Figure S45 and Figure S46.

3 Computational methodology (density functional theory)

All *ab initio* and density functional theory (DFT) calculations were performed in ORCA 4.2.1.[8] The B3LYP functional was used in conjunction with the Becke-Johnson damped D3 dispersion correction[9] (D3BJ) to optimise geometries and obtain harmonic frequencies. For all species in this study, we verified that our conformations were true minima possessing no imaginary frequencies. Improved single point energies were then calculated with DLPNO-CCSD(T).

For B3LYP calculations, the def2-TZVP basis set was used,[10] in conjunction with the RIJCOSX approximation and a def2/J auxiliary basis set.[11] To minimise potential numerical artefacts, 'Grid7' and 'GridX7' was used in conjunction with the 'NoFinalGrid' keyword. To avoid reported issues with RI-integral pre-screening for analytical hessian calculations, pre-screening was turned off for frequency calculations. For DLPNO-CCSD(T) calculations,[12] the cc-pVTZ basis set was used,[13,14] in conjunction with the RIJCOSX approximation and both def2/J and cc-pVTZ/C auxiliary basis sets.[11,15] In addition, 'TightPNO' cut-offs were applied. 'VeryTight' SCF convergence criteria was used for all ground-state calculations. Free energies of solvation (in acetonitrile) were calculated using the SMD solvent model.[16]

For STEOM-DLPNO-CCSD calculations,[17] the cc-pVTZ basis set was used,[13,14] in conjunction with the RIJCOSX approximation and both def2/J and cc-pVTZ/C auxiliary basis sets.[11,15] For these excited state calculations, solvent corrections were calculated in acetonitrile using the CPCM solvent model.[18] In addition, 'Tight' SCF convergence criteria were used in conjunction with 'GridX7'.

Raw Energies

Table S23. Raw energies for all the species investigated in this study. Unless specified, units are in Hartree.

Species	DLPNO-CCSD(T)/ cc-pVTZ	B3LYP-D3BJ/ def2-TZVP	ZPVE	TC	S	ΔG_{solv}(kcal/mol)	H_{gas}	G_{gas}	G_{sol}
EtherOMB.anti	-498.555364	-499.347721	0.169773	0.010478	0.046770	-1.275	-498.374169	-498.420940	-498.419953
EtherOMB.syn	-498.548755	-499.341786	0.169603	0.010565	0.046983	-1.332	-498.367643	-498.414626	-498.413730
ThioEtherOMB.anti.eclipsed	-821.173603	-822.295516	0.165876	0.011146	0.048389	-1.957	-820.995636	-821.044025	-821.044125
ThioEtherOMB.syn.eclipsed	-821.173116	-822.296168	0.166218	0.010889	0.047739	-1.967	-820.995065	-821.042805	-821.042921
ThioEtherOMB.anti.staggered	-821.173504	-822.294311	0.165822	0.011189	0.048514	-2.102	-820.995548	-821.044062	-821.044393
ThioEtherOMB.syn.staggered	-821.168158	-822.289604	0.165827	0.011226	0.048690	-1.884	-820.990161	-821.038851	-821.038835

Optimised Gas-Phase Geometries

EtherOMB.anti

```
21
0 1
 C   -3.17334457208300    1.59259540218066    0.01019737749739
 C   -3.01867733951233    0.20936151295853    0.00169150052966
 C   -1.73867298995964   -0.32593783779638   -0.01296619515417
 C   -0.62240657702255    0.49560427352055   -0.01920498433210
 C   -0.75047741325010    1.88282711560442   -0.01092142284121
 C   -2.04432189854074    2.44789159449434    0.00405301467991
 O   -4.39068952021607    2.19405235614852    0.02458169244179
 H   -3.87546931196656   -0.44622976959771    0.00634167081981
 H   -1.61509398845983   -1.40176630152061   -0.01959917735021
 H    0.36743684911825    0.05814893465945   -0.03069896080234
 C   -2.27378333770687    3.90479238595956    0.01380074757788
 O   -1.40768901752208    4.75644157835466    0.01001406719557
 H   -3.33414510340927    4.20183705838716    0.02543133359916
 C    0.49644879710450    2.72089833381508   -0.01830754645465
 C   -5.55817082547291    1.38796069219472    0.03153306669195
 H    1.37557249878161    2.07639116253228   -0.02967197040039
 H    0.54322756127513    3.37414546672823    0.85324709487258
 H    0.52712671021488    3.38278620980981   -0.88404565018097
 H   -6.39581744410923    2.08093403156996    0.04273671039776
 H   -5.59835996888661    0.75411807919152    0.92157721354785
 H   -5.61502310837657    0.76285772080524   -0.86375958233527
```

EtherOMB.syn

```
21
0 1
 C   -3.21154142830756    1.58601838999229    0.01665961167107
 C   -3.02619765875074    0.20212833773620    0.00381637055856
 C   -1.74249310574919   -0.31764381670923   -0.01839929336464
 C   -0.63612066340229    0.51888789559360   -0.02816378579235
 C   -0.79223261940256    1.90096704937902   -0.01574186795526
 C   -2.09085194351374    2.45573980730586    0.00703207117342
 O   -4.42986876739418    2.15359471542698    0.03822799180886
 H   -3.87362704079402   -0.46549207729149    0.01107081894045
 H   -1.60578677394590   -1.39186020346076   -0.02823265444700
 H    0.35992841135571    0.09624075848188   -0.04561816402103
 C   -2.23991836131913    3.92465677296787    0.02031504447057
 O   -3.26977386405326    4.56028473473295    0.04030251467380
 H   -1.27674682153548    4.46687771745398    0.01063883856497
 C    0.45358659399267    2.75522530180174   -0.02746052901362
 C   -5.58258871951519    1.32713311034557    0.04894957061531
 H    1.33596722831707    2.11681122947209   -0.04470573840507
 H    0.52286887819152    3.39306669730704    0.85552646610810
 H    0.49763468815683    3.40568210257693   -0.90284334448770
 H   -6.42888582329863    2.00895519782751    0.06585986624094
 H   -5.60909830193483    0.69045694132924    0.93784978894524
 H   -5.63469390709708    0.70297933773073   -0.84765357628462
```

ThioetherOMB.anti.eclipsed

```
21
0 1
 C   -2.88673929695520    1.75396809269072    0.01433936583007
 C   -2.78883920561134    0.36417593087549    0.00236216800315
 C   -1.54332431403327   -0.24392423135156   -0.02114932044624
 C   -0.38369135000510    0.51448279472657   -0.03301929600139
 C   -0.43826570452342    1.90535321264361   -0.02175943420592
 C   -1.70231871809307    2.53736653528807    0.00207099865023
 S   -4.46732133703906    2.56238325381486    0.04497030030387
 H   -3.67183020207669   -0.25423949590940    0.01122426986947
 H   -1.48180518125693   -1.32517720278941   -0.03020893672214
 H    0.58154184202144    0.02514829726763   -0.05131490157332
 C   -1.82727436587221    4.00548062903701    0.01449487376815
 O   -0.91613360657341    4.80507962818052    0.00757071848329
 H   -2.86759427062590    4.37929104882826    0.03161932668785
 C    0.85403844535465    2.67236778650668   -0.03561670081251
 C   -5.64446104767752    1.19051918647000    0.05050234306351
 H    1.69446850508726    1.97834904831443   -0.05346362327623
```

H 0.94454733355285 3.31954372148138 0.83690730684003
H 0.91830603272569 3.33239907929967 -0.90083241501713
H -6.62355747910877 1.66729683503983 0.06827194541774
H -5.53794558301021 0.56933699737284 0.93876656704872
H -5.56324049627977 0.58397885221277 -0.85046555591120

ThioetherOMB.syn.eclipsed

21
0 1
C -2.91868449408510 1.77109456763187 0.01404629957366
C -2.80380504355064 0.37633465582162 0.00157099498886
C -1.56026179647869 -0.22833456304748 -0.02091752369988
C -0.39663426859721 0.53044340669024 -0.03179740730532
C -0.46328140774119 1.91613492243904 -0.02032571322137
C -1.72782866788837 2.54784109107185 0.00263949909364
S -4.49066910810234 2.56580523372595 0.04292902612927
H -3.68260780513276 -0.24706772874187 0.00937583122772
H -1.49821940956774 -1.30960121487475 -0.03008238060208
H 0.56884069412589 0.04158898501458 -0.04937685007322
C -1.79161067025730 4.00986959747875 0.01440851458943
O -2.81820129876941 4.65999133477498 0.03412625319033
H -0.82083471750972 4.53257080889405 0.00378983149467
C 0.82816747271952 2.69842918295833 -0.03303470426963
C -5.63719759478380 1.15908156398766 0.04906643391510
H 1.67424783417913 2.01262523185757 -0.04992294848529
H 0.93255785164862 3.33187439727692 0.84982194445782
H 0.90761012181246 3.34444578216655 -0.90937398519198
H -6.62529886947737 1.61799295733475 0.06562999664330
H -5.52554756333046 0.53808477765404 0.93752922057080
H -5.54994125921348 0.55014500988532 -0.85041233302584

ThioetherOMB.anti.staggered

21
0 1
C -2.77410163477235 2.02379987386737 0.31334882915143
C -2.83910809006724 0.64844552997046 0.11905512642495
C -1.67813269062682 -0.06944430171961 -0.12855593771812
C -0.45845751311422 0.58579888539320 -0.21277267291157
C -0.35614184293983 1.96358468140151 -0.03080769360581
C -1.53090463783896 2.69110453068564 0.26496322084993
S -4.32014808752355 2.86419835167226 0.62535465471750
H -3.80070188160092 0.15475084134084 0.14899237678657
H -1.72773286000462 -1.14119117558356 -0.27447662896813
H 0.43994030514152 0.01944334956552 -0.42452736875325
C -1.47864896931233 4.13074005816140 0.60177799290969
O -0.55866595376935 4.88289277119138 0.36418766109291
H -2.37365215190161 4.49517223861844 1.13636866299211
C 0.99841049349069 2.60752741332966 -0.13397873782715
C -4.45507193580665 3.93192450408410 -0.84342402551973
H 1.76264906173732 1.84306065660091 -0.27448521175984
H 1.23787988849306 3.18886021767569 0.75681471925611
H 1.04172319033672 3.30874286947308 -0.96736971269788
H -5.43857711974767 4.39746404837035 -0.78704116681701
H -4.39032964865372 3.33572563175150 -1.75186133769337
H -3.69413792151945 4.71057902414984 -0.85201274990936

ThioetherOMB.syn.staggered

21
0 1
C -0.21918711616195 1.81429904931886 0.46001847344943
C -0.45816603165583 0.54179081969576 -0.06115320621191
C -1.73719049496014 0.14756225955193 -0.41618826902164
C -2.80520594509608 1.01631233377742 -0.24197151324316
C -2.60740756765022 2.30291390656590 0.24440404969822
C -1.29852244979326 2.72396463235007 0.58252809146569
S 1.48495410010697 2.17029246859560 0.81858973426787
H 0.37412549472241 -0.14102997660104 -0.16197167121824
H -1.90520384950188 -0.84773289985628 -0.80800531848424
H -3.80849294830663 0.69392367884705 -0.49088970508756
C -1.11431330715820 4.14547276600903 0.94003765901044
O -0.13050978649683 4.65557847299145 1.42689319912764

H	-1.97808002814404	4.78526400121755	0.68863125870232
C	-3.81323154313585	3.19990320512066	0.39943284821404
C	1.48779682253770	2.45888700882969	2.61209408945092
H	-4.72240014346216	2.63327282079195	0.20196039998016
H	-3.79274250726179	4.03923183076475	-0.29950042868465
H	-3.89106057864492	3.61514311301130	1.40502896669341
H	2.53936240712498	2.49528360107610	2.89630515969435
H	1.00906999573949	1.62676525828626	3.12579351450077
H	1.00611547719825	3.40172164965591	2.85121266769611

Other Supplementary Data and Files

The STL files for the 3D printed parts as well as files containing the source code of algorithms included in this work are available online as supplementary datasets. Note: The photoreactor was manufactured by resin printing (stereolithography), while the manual shutter and the detector scaffold were manufactured by FDM printing (Fused Deposition Modelling). The manual shutter was treated with sandpaper to allow for a smooth fit into the photoreactor.

Author Contributions

J.P.M. (Jan Philipp Menzel) was responsible for the experimental conceptualization, writing of source code, running of simulations, conducting experiments and writing of the original manuscript. B.B.N. (Benjamin Noble) was responsible for wavefunction and density functional theory calculations. C.B.-K. (Christopher Barner-Kowollik) and J.P.B. (James P. Blinco) motivated the study, supervised the project, discussed the data, edited the manuscript and provided the conceptual framework for visible light photochemistry. AIl authors have given approval to the final version of the manuscript.

References

1 Menzel, J. P. *et al.* Light-Controlled Orthogonal Covalent Bond Formation at Two Different Wavelengths. *Angew. Chem. Int. Ed.* **58**, 7470-7474, (2019).
2 Menzel, J. P. *et al.* Wavelength Dependence of Light-Induced Cycloadditions. *J. Am. Chem. Soc.* **139**, 15812-15820, (2017).
3 Fast, D. E. *et al.* Wavelength-Dependent Photochemistry of Oxime Ester Photoinitiators. *Macromolecules* **50**, 1815-1823, (2017).
4 Tuten, B. T., Menzel, J. P., Pahnke, K., Blinco, J. P. & Barner-Kowollik, C. Pyreneacyl sulfides as a visible light-induced versatile ligation platform. *Chem. Commun.* **53**, 4501-4504, (2017).
5 Feist, F., Menzel, J. P., Weil, T., Blinco, J. P. & Barner-Kowollik, C. Visible Light-Induced Ligation via o-Quinodimethane Thioethers. *J. Am. Chem. Soc.* **140**, 11848-11854, (2018).
6 Gegenhuber, T. *et al.* Fusing Light-Induced Step-Growth Processes with RAFT Chemistry for Segmented Copolymer Synthesis: A Synergetic Experimental and Kinetic Modeling Study. *Macromolecules* **50**, 6451-6467, (2017).
7 Frisch, H. *et al.* Photochemistry in Confined Environments for Single-Chain Nanoparticle Design. *J. Am. Chem. Soc.* **140**, 9551-9557, (2018).
8 Neese, F. The ORCA program system. *WIREs Computational Molecular Science* **2**, 73-78, (2012).
9 Grimme, S., Ehrlich, S. & Goerigk, L. Effect of the damping function in dispersion corrected density functional theory. *Journal of Computational Chemistry* **32**, 1456-1465, (2011).
10 Weigend, F. & Ahlrichs, R. Balanced basis sets of split valence, triple zeta valence and quadruple zeta valence quality for H to Rn: Design and assessment of accuracy. *Physical Chemistry Chemical Physics* **7**, 3297-3305, (2005).
11 Weigend, F. Accurate Coulomb-fitting basis sets for H to Rn. *Physical Chemistry Chemical Physics* **8**, 1057-1065, (2006).
12 Riplinger, C., Sandhoefer, B., Hansen, A. & Neese, F. Natural triple excitations in local coupled cluster calculations with pair natural orbitals. *The Journal of Chemical Physics* **139**, 134101, (2013).
13 Jr., T. H. D. Gaussian basis sets for use in correlated molecular calculations. I. The atoms boron through neon and hydrogen. *The Journal of Chemical Physics* **90**, 1007-1023, (1989).
14 Woon, D. E. & Jr., T. H. D. Gaussian basis sets for use in correlated molecular calculations. III. The atoms aluminum through argon. *The Journal of Chemical Physics* **98**, 1358-1371, (1993).
15 Weigend, F., Köhn, A. & Hättig, C. Efficient use of the correlation consistent basis sets in resolution of the identity MP2 calculations. *The Journal of Chemical Physics* **116**, 3175-3183, (2002).
16 Marenich, A. V., Cramer, C. J. & Truhlar, D. G. Universal Solvation Model Based on Solute Electron Density and on a Continuum Model of the Solvent Defined by the Bulk Dielectric Constant and Atomic Surface Tensions. *The Journal of Physical Chemistry B* **113**, 6378-6396, (2009).
17 Berraud-Pache, R., Neese, F., Bistoni, G. & Izsák, R. Unveiling the Photophysical Properties of Boron-dipyrromethene Dyes Using a New Accurate Excited State Coupled Cluster Method. *Journal of Chemical Theory and Computation* **16**, 564-575, (2020).
18 Barone, V. & Cossi, M. Quantum Calculation of Molecular Energies and Energy Gradients in Solution by a Conductor Solvent Model. *The Journal of Physical Chemistry A* **102**, 1995-2001, (1998).
