# Supplementary material for: Predicting wavelength-dependent photochemical reactivity and selectivity
Source: Nat Commun. 2021 Mar 16;12:1691. doi: 10.1038/s41467-021-21797-x (PMC7966369; doi:10.1038/s41467-021-21797-x)
Supplement: Supplementary file 3 — Reporting Summary [file 41467_2021_21797_MOESM3_ESM.pdf]

nature research

Corresponding author(s): James P. Blinco; Christopher Barner-Kowollik

Last updated by author(s): Jan 25, 2021

Reporting Summary

Nature Research wishes to improve the reproducibility of the work that we publish. This form provides structure for consistency and transparency in reporting. For further information on Nature Research policies, see our Editorial Policies and the Editorial Policy Checklist.

Statistics

For all statistical analyses, confirm that the following items are present in the figure legend, table legend, main text, or Methods section.

n/a	Confirmed	
☐	☒	The exact sample size (n) for each experimental group/condition, given as a discrete number and unit of measurement
☐	☒	A statement on whether measurements were taken from distinct samples or whether the same sample was measured repeatedly
☒	☐	The statistical test(s) used AND whether they are one- or two-sided<br>*Only common tests should be described solely by name; describe more complex techniques in the Methods section.*
☒	☐	A description of all covariates tested
☐	☒	A description of any assumptions or corrections, such as tests of normality and adjustment for multiple comparisons
☐	☒	A full description of the statistical parameters including central tendency (e.g. means) or other basic estimates (e.g. regression coefficient) AND variation (e.g. standard deviation) or associated estimates of uncertainty (e.g. confidence intervals)
☒	☐	For null hypothesis testing, the test statistic (e.g. F, t, r) with confidence intervals, effect sizes, degrees of freedom and P value noted<br>*Give P values as exact values whenever suitable.*
☒	☐	For Bayesian analysis, information on the choice of priors and Markov chain Monte Carlo settings
☒	☐	For hierarchical and complex designs, identification of the appropriate level for tests and full reporting of outcomes
☒	☐	Estimates of effect sizes (e.g. Cohen's d, Pearson's r), indicating how they were calculated

Our web collection on statistics for biologists contains articles on many of the points above.

Software and code

Policy information about availability of computer code

Data collection	NMR data was collected with Bruker ICON-NMR software / Topspin 3.5pl7. Mass spectrometry data was collected with ThermoFischer software ExactiveTune / Xcalibur. UV Vis spectra were collected with Shimadzu software UVProbe 2.43. LED power measurements were carried out with a Coherent FieldMate Laser Power Meter, Model PM2. LED emission spectra were measured with an Ocean Optics Miniature Spectrometer FLAME-T-UV-VIS.
Data analysis	Python source code is publicly available at: https://github.com/jphmenzel/jpmphotochem. The STL files for the 3D printed parts as well as files containing the source code of algorithms included in this work are available online as supplementary datasets.<br>NMR data was analyzed with MestReNova and OriginPro9.1G. Mass spectrometry data was processed with Xcalibur, mMass – Open Source Mass Spectrometry Tool, Microsoft Excel and OriginPro 9.1G. LED emission spectra were collected with OceanView 1.6.7.

For manuscripts utilizing custom algorithms or software that are central to the research but not yet described in published literature, software must be made available to editors and reviewers. We strongly encourage code deposition in a community repository (e.g. GitHub). See the Nature Research guidelines for submitting code & software for further information.

Data

Policy information about availability of data

All manuscripts must include a data availability statement. This statement should provide the following information, where applicable:

- Accession codes, unique identifiers, or web links for publicly available datasets
- A list of figures that have associated raw data
- A description of any restrictions on data availability

The data that support the findings of this study are available from the corresponding author upon reasonable request. Source data for figures 2 – 8 and STL files for 3D printed parts of the photoreactor are provided online with the paper.

Field-specific reporting

Please select the one below that is the best fit for your research. If you are not sure, read the appropriate sections before making your selection.

[x] Life sciences [ ] Behavioural & social sciences [ ] Ecological, evolutionary & environmental sciences

For a reference copy of the document with all sections, see nature.com/documents/nr-reporting-summary-flat.pdf

Life sciences study design

All studies must disclose on these points even when the disclosure is negative.

Sample size	Experiments were either carried out as single experiments or in triplicate.
Data exclusions	No data are excluded from the analysis of replicates and reported experiments. Previous, early experiments without the 3D-printed photoreactor, which are not relevant to the study, are not shown, as they were carried out in a less reproducible way due to the variability in the light dose that is delivered in an experiment with an insufficiently controlled irradiation geometry.
Replication	Experiments for the determination of wavelenth-dependent quantum yields were carried out in triplicate, all replicates are included. Experiments regarding the concentration dependence were single experiments, but the trend was investigated at different wavelengths and using different analytical methods to confirm the observation. LED irradiation experiments regarding the prediction of time-dependent conversion, were carried out in triplicate, with all replicates included. LED experiments regarding the selectivity between reaction channels were carried out as single experiments or in triplicate with all replicates included. Every results is found to be reproducible.
Randomization	This is not relevant, as the chemical reactivity of molecules is studied. The instrumental analysis is independent from human bias, as the samples were evaluated equally.
Blinding	Blinding was not possible and is not relevant, as due to the precise parametrization of the experiment, no change of the outcome of the experiment is possible without changing defined experimental parameters. Experimental results are reported as they are observed.

Reporting for specific materials, systems and methods

We require information from authors about some types of materials, experimental systems and methods used in many studies. Here, indicate whether each material, system or method listed is relevant to your study. If you are not sure if a list item applies to your research, read the appropriate section before selecting a response.

Materials & experimental systems

n/a	Involved in the study
[x]	[ ] Antibodies
[x]	[ ] Eukaryotic cell lines
[x]	[ ] Palaeontology and archaeology
[x]	[ ] Animals and other organisms
[x]	[ ] Human research participants
[x]	[ ] Clinical data
[x]	[ ] Dual use research of concern

Methods

n/a	Involved in the study
[x]	[ ] ChIP-seq
[x]	[ ] Flow cytometry
[x]	[ ] MRI-based neuroimaging
